# Single-Cell (Meta-)Genomics of a Dimorphic *Candidatus* Thiomargarita nelsonii Reveals Genomic Plasticity

**DOI:** 10.3389/fmicb.2016.00603

**Published:** 2016-05-03

**Authors:** Beverly E. Flood, Palmer Fliss, Daniel S. Jones, Gregory J. Dick, Sunit Jain, Anne-Kristin Kaster, Matthias Winkel, Marc Mußmann, Jake Bailey

**Affiliations:** ^1^Department of Earth Sciences, University of MinnesotaMinneapolis, MN, USA; ^2^Biotechnology Institute, University of MinnesotaSt. Paul, MN, USA; ^3^Department of Earth and Environmental Sciences, University of MichiganAnn Arbor, MI, USA; ^4^German Collection of Microorganisms and Cell Cultures, Leibniz Institute DSMZBraunschweig, Germany; ^5^Helmholtz Centre Potsdam, GFZ German Research Centre for GeosciencesPotsdam, Germany; ^6^Max Planck Institute for Marine MicrobiologyBremen, Germany

**Keywords:** *Thiomargarita*, single-cell genomics, arsenite oxidation, intron, mobile genetic elements, genome instability, miniature inverted-repeat transposable elements, metacaspase

## Abstract

The genus *Thiomargarita* includes the world's largest bacteria. But as uncultured organisms, their physiology, metabolism, and basis for their gigantism are not well understood. Thus, a genomics approach, applied to a single *Candidatus* Thiomargarita nelsonii cell was employed to explore the genetic potential of one of these enigmatic giant bacteria. The *Thiomargarita* cell was obtained from an assemblage of budding *Ca*. T. nelsonii attached to a provannid gastropod shell from Hydrate Ridge, a methane seep offshore of Oregon, USA. Here we present a manually curated genome of Bud S10 resulting from a hybrid assembly of long Pacific Biosciences and short Illumina sequencing reads. With respect to inorganic carbon fixation and sulfur oxidation pathways, the *Ca*. T. nelsonii Hydrate Ridge Bud S10 genome was similar to marine sister taxa within the family *Beggiatoaceae*. However, the Bud S10 genome contains genes suggestive of the genetic potential for lithotrophic growth on arsenite and perhaps hydrogen. The genome also revealed that Bud S10 likely respires nitrate via two pathways: a complete denitrification pathway and a dissimilatory nitrate reduction to ammonia pathway. Both pathways have been predicted, but not previously fully elucidated, in the genomes of other large, vacuolated, sulfur-oxidizing bacteria. Surprisingly, the genome also had a high number of unusual features for a bacterium to include the largest number of metacaspases and introns ever reported in a bacterium. Also present, are a large number of other mobile genetic elements, such as insertion sequence (IS) transposable elements and miniature inverted-repeat transposable elements (MITEs). In some cases, mobile genetic elements disrupted key genes in metabolic pathways. For example, a MITE interrupts *hupL*, which encodes the large subunit of the hydrogenase in hydrogen oxidation. Moreover, we detected a group I intron in one of the most critical genes in the sulfur oxidation pathway, *dsrA*. The *dsrA* group I intron also carried a MITE sequence that, like the *hupL* MITE family, occurs broadly across the genome. The presence of a high degree of mobile elements in genes central to *Thiomargarita*'s core metabolism has not been previously reported in free-living bacteria and suggests a highly mutable genome.

## Introduction

The family *Beggiatoaceae* include the largest known free-living bacteria with some marine *Thiomargarita* spp. achieving millimetric cell diameters (Bailey et al., 2009; Salman et al., 2011). These bacteria are chemolithotrophs that obtain energy for metabolism from the oxidation of reduced sulfur species. *Thiomargarita* spp. are thought to primarily use the oxidation of electron donors available in the sediment pore waters to fuel carbon fixation. The terminal electron acceptors used in these reactions can vary. Besides oxygen, nitrate can be used as a terminal electron acceptor in large, vacuolated sulfur bacteria under anoxic conditions, and *Thiomargarita* spp. allocates up to 90% of its volume for intracellular nitrate storage (Schulz and Jørgensen, 2001). *Thiomargarita* spp. have also been shown to accumulate intracellular elemental sulfur inclusions that serve as intermediates in the oxidation of hydrogen sulfide to sulfate, to provide the cell with electron donors when access to sulfide is limited (Schulz et al., 1999). Prior research has also demonstrated that *Thiomargarita* spp. are capable of accumulating phosphate intracellularly as long polyphosphate (poly-p) polymers. The hydrolysis of this polyphosphate, and concomitant release of phosphate into pore water has been linked to the formation of large phosphorite deposits in the seafloor and the subsurface (Schulz and Schulz, 2005; Bailey et al., 2006, 2013; Goldhammer et al., 2010; Crosby and Bailey, 2012; Dale et al., 2013). However, the stimuli and mechanisms for polyphosphate accumulation and release of inorganic phosphorous have not been fully elucidated (Brock and Schulz-Vogt, 2011).

**Figure 1:**
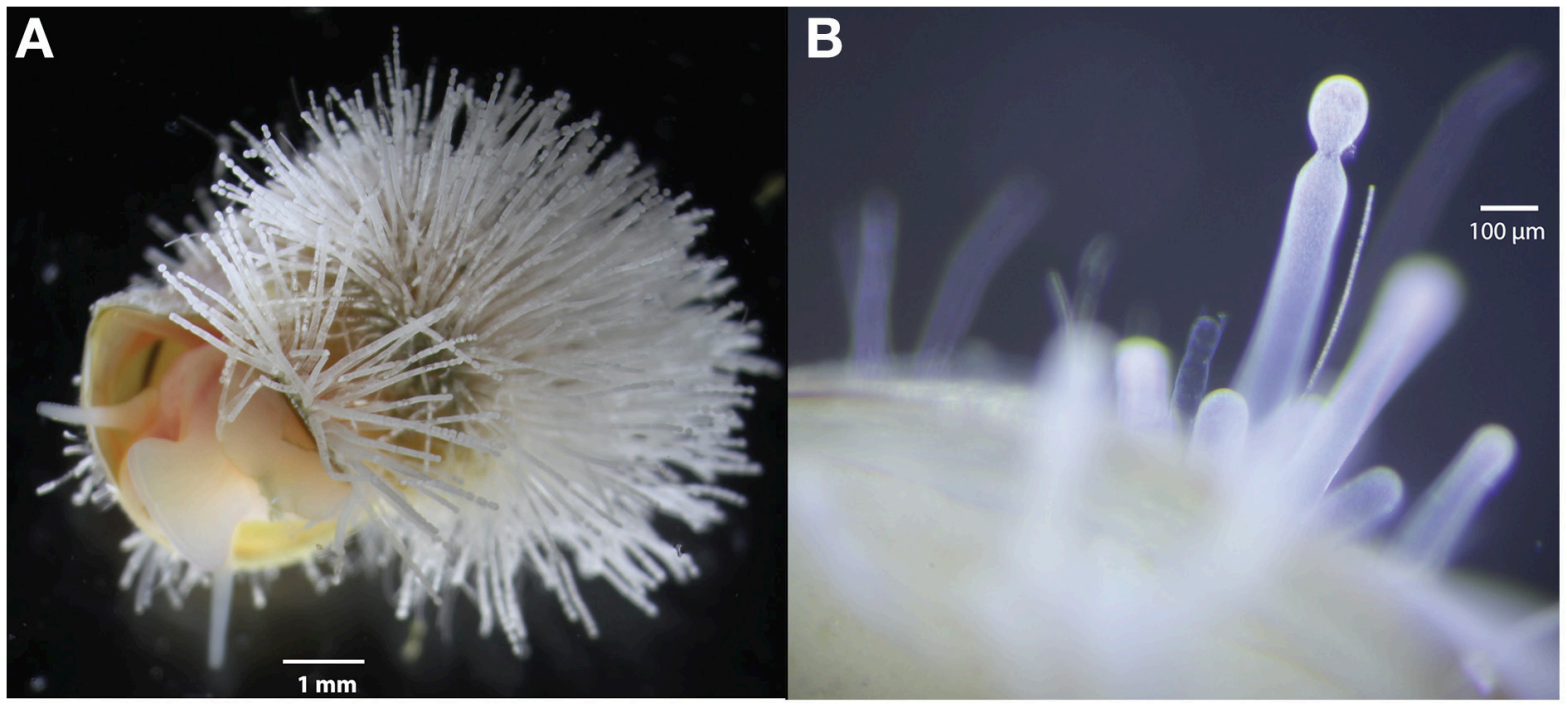
**(A)** *Provanna* sp. snail with attached *Thiomargarita* epibiont community. **(B)** An elongated *Thiomargarita* morphotype attached to provannid snail that is budding from the distal end. Bud S10 was derived from this population of actively budding daughter cells.

Marine cold seeps are sites where elevated hydrocarbon fluxes fuel the production of sulfide via the anaerobic oxidation of methane and sulfate reduction (Boetius et al., 2000). Dimorphic *Thiomargarita* ecotypes of *Candidatus* Thiomargarita nelsonii initially discovered at seeps along the Costa Rican Margin were later observed at Hydrate Ridge. These ecotypes are commonly found attached to substrates, particularly the shells of provannid snails, Figure 1A. These attached cells appeared to undergo a dimorphism (elongate vs. budding) in their life cycle, wherein *Thiomargarita* elongated almost a millimeter in length, and budded spherical daughter cells from the distal end (Figure 1B; Bailey et al., 2011). Several of the gastropod samples collected showed a distinct epibiont community resembling morphotypes similar to *Ca*. T. nelsonii as well as *Marithrix* sp. and *Leucothrix* sp. (Salman et al., 2013). Here we report on a new draft genome assembly of a single cell of *Ca*. T. nelsonii Hydrate Ridge budding from an attached *Thiomargarita* cell that we refer to as “Bud S10.” Despite washing the bud, several adherent bacteria were not removed, thus a metagenome was assembled using Illumina and Pacific Biosciences sequencing reads that were binned and manually-curated to produce the genome presented here.

The draft genome revealed many comparable metabolic pathways to those found in sister taxa *Ca*. Maribeggiatoa Guaymas Basin orange filament (BOGUAY) (MacGregor et al., 2013a) and in a *Ca*. T. nelsonii phylotype from Namibia (Winkel et al., in review). However, we found a number of surprising genomic features, including genes that suggest the potential for lithotrophic growth using arsenite as an electron donor. In addition, we found genes encoding two complete nitrate reduction pathways, one that terminates in N_2_ and another that produces ${\text{NH}}_{4}^{+}$, as predicted by metabolic studies of giant marine sulfur bacteria (Otte et al., 1999; Høgslund et al., 2009; Prokopenko et al., 2013), but incomplete in previously sequenced genomes (Mußmann et al., 2007; MacGregor et al., 2013a). Surprisingly, the genome displayed a high number of mobile genetic elements that we describe here. These mobile elements include group I and II introns, transposons, and miniature inverted-repeat transposable elements (MITEs). Interestingly, key genes in sulfur and hydrogen oxidation pathways were disrupted by mobile elements. Furthermore, we describe a new form of molecular symbiosis between a group I intron and MITE.

## Materials and methods

### Site description and sample collection

During a research expedition on board the R/V *Atlantis* (AT18_1) to Hydrate Ridge North (44° 40.02687′ N 125° 5.99969′ W), Cascadian margin, off the coast of Oregon, USA the remotely operated vehicle *Jason* collected methane seep samples containing provannid gastropods. The gastropod exteriors hosted dense biofilms on the posterior surface of their shells. These biofilms included attached *Thiomargarita*-like bacteria similar to those described by Bailey et al. (2011).

These snails were fixed shipboard in a 1:1 mixture of sterile ethanol and Instant Ocean® (Spectrum Brands, USA) and then stored at −20°C for molecular analyses.

### Genomic DNA amplification, sequencing, and assembly

An ethanol fixed gastropod and attached community was examined under an Olympus SZX-16 stereo microscope. Large bacterial morphotypes were individually removed from the host via a pipet. The genome was obtained from a single *Ca*. T. nelsonii bud (sample S10) that detached from an elongated attached *Ca*. T. nelsonii cell. The cell was placed in a 40 μm sterile cell strainer (BD Biosciences, San Diego, CA) and rinsed seven times in DNA-free water with 3.5% NaCl and 50% ethanol. Despite our best effort to clean the *Thiomargarita* cell, some adherent bacterial epibionts were not removed. Whole genome amplification was performed using a RePLI-g midi-kit (Qiagen, USA) according to the manufacturer's instructions. DNA sequencing was performed on a Illumina HiSeq 2000 (CASAVA 1.8), which yielded 85 million 100 base pair (bp) paired end reads with 300 bp inserts (Truseq v1 chemistry). A previous metagenome assembly was performed using these Illumina sequencing reads (Fliss, 2014). Here, we included additional DNA sequencing using 12 Pacific Biosciences RS SMRT cells prepared with P4-C2 chemistry. Seven SMRT cells contained fragmented DNA of ~600 bases for obtaining high quality circular consensus sequencing (CCS) reads and five SMRT cells for continuous long read (CLR) sequencing of unfragmented DNA. A combination of cutadapt (Martin, 2011), Sickle (v.1.29) (Joshi and Fass, 2011) and Prinseq-lite (v.0.20.4) (Schmieder and Edwards, 2011) were used to error correct the Illumina reads (sliding window q20 at the 3′ end, sequences containing N's removed, average read q30, de-replication of six or more exact duplicates and minimum read length of 50 bases). The PacBio CCS reads were filtered (RS_Subreads.1) using the SMRT Analysis (v.2.20) software package (http://www.pacb.com/devnet/) and concatenated with Illumina unpaired reads generated during the quality filtering and trimming step. Additionally, sequence data from all 12 SMRT cells were filtered and were used for scaffolding and were flagged as “uncorrected PacBio” reads. A hybrid assembly was performed using SPAdes 3.1 with “assembler only” mode (no pre-assembly error correction) with kmer sizes of 21, 35, 55, and 75 and with post-assembly mismatch correction. The final assembled metagenome was 31.68 Mbp (3326 contigs) with an N50 value of 67,259 bp.

### Community assessment, tetramer-frequency binning, and curation

In preparation for tetranucleotide binning, rRNA gene sequences in the metagenomes were analyzed to identify constituent strains in the dataset. Putative rRNA gene fragments in the metagenome were first identified by comparing unassembled quality-filtered Illumina reads against the Silva small subunit (SSU) rRNA reference database (version 115) (Quast et al., 2013) using BLASTN (McGinnis and Madden, 2004). SSU rRNA gene sequence matches were then extracted from the Illumina dataset and assembled using EMIRGE (Miller et al., 2011). The EMIRGE assembly yielded five 16S rRNA sequences. The best BLASTN matches for these five sequences were *Pseudoalteromonas* sp. M12-11A FN377706 (representing 41% of rRNA genes according to EMIRGE), *Colwellia* sp. BCw110 FJ889596 (26%), an uncultured *Colwellia* AY375054 (18%), *Ca*. T. nelsonii (9%), and *Neptuniibacter* sp. CAR-SF AB086227 (5%). Separately, a 16S rRNA gene and intergenic region with 100% identity to clone *Ca*. T. nelsonii HYR001 (GenBank accession number HF954113) (Salman et al., 2013) was amplified and sequenced from the RePLI-g-processed DNA using *Thiomargarita*-specific PCR primers (VSO233F—ITSReub) (Salman et al., 2011).

A tetramer-frequency based Emergent Self-Organizing Map (tetra-ESOM) was constructed based on the contigs generated, following the protocol outlined in Ultsch and Mörchen (2005), Dick et al. (2009). The genomes of the following strains were included in the tetra-nucleotide training and ESOM binning: *Candidatus* Beggiatoa sp. Orange Guaymas (NCBI project ID: PRJNA19285, Locus Tag BOGUAY), *Colwellia piezophila* BAA-637 (PRJNA182419, F580), *Colwellia psychrerythraea* 34H (PRJNA275, CPS), *Neptuniibacter caesariensis* MED92 (PRJNA13561, MED92), *Pseudoalteromonas* SM9913 (PRJNA39311, PSM), and *Candidatus* Thiomargarita sp. Thio36 (PRJNA79059, Thi036) from Namibian coastal upwelling sediments. Only contigs greater than 2 kb were used in the tetra-ESOM analyses. The network was trained with a K-Batch algorithm, 170 rows and 354 columns (~60,180 neurons), and a starting radius value of 50. In total, 543 metagenome contigs overlapped or neighbored the genomes of BOGUAY and Thio36 and were selected for the *Thiomargarita* bin. Subsequently, 32 contigs were removed from the bin based on coverage below 20x and/or less than 10% of the contig fell within the bin. Additional analyses using kmer coverage on the binned genome submitted to IMG/ER and a kmer analysis (vs. 4.5) was performed (oligomer size 4, fragment window 5000, fragment step 500) in addition to manual curation and comparison to the *Ca*. T. nelsonii Thio36 genome. These analyses resulted in the removal of 72 additional contigs including high coverage contigs that contains plasmid related genes.

### Annotation and bioinformatics

Annotation was performed by the IMG/ER gene prediction pipeline (Markowitz et al., 2009). Since IMG was the primary tool used for genome analyses, IMG gene and contig notations are used herein. After assessing the genome, we found that several genes were absent from the hybrid assembly (contigs greater than 1500 bp) but present in the original non-hybrid assembly (IMG Gold ID Ga0097846). The genes were located on contigs Ga0097846_10092 (8217 bp), Ga0097846_10099 (2891 bp), Ga0097846_11126 (14,449 bp), Ga0097846_11134 (10,846 bp), Ga0097846_11318 (11,275 bp), (Ga0097846_11205 (7845 bp), and Ga0097846_12056 (5245 bp). These genes were included in the assessment of metabolic pathways below. Alignments and comparison of group I Intron containing sequences was performed using UGENE (Okonechnikov et al., 2012). Repetitive sequences with 15 or more occurrences were identified using the RepeatModeler (Smit and Hubley, 2008-2015). Group II introns were identified using the Database for Bacterial group II Introns (Candales et al., 2011). Repetitive elements identified by RepeatModeler and putative introns where compared with sequences in the RNA families database (Rfam) (Nawrocki et al., 2014) and examined for protein-encoding domains with the NCBI Conserved Domain Database (CDD) (Marchler-Bauer et al., 2014). MITE sequences were compared with IS elements deposited in ISfinder database (https://www-is.biotoul.fr) (Siguier et al., 2006). All BLASTN analyses against Bud S10's genome were performed with a cutoff *e*-value of 10e^−5^.

Arsenite oxidoreductase (AioA) peptide sequences were aligned with the Expresso algorithm in T-Coffee using default parameters (Armougom et al., 2006), and the alignment trimmed with the “automated1” option in TrimAL (Capella-Gutiérrez et al., 2009) at the Phylomon2 online workbench (Sánchez et al., 2011). The final alignment length was 885 amino acid positions. Maximum likelihood analyses were performed with RAxML version v 8.0.24 (Stamatakis, 2006) with 1000 rapid bootstrap replicates. For maximum likelihood analysis, the LG amino acid substitution model (Le and Gascuel, 2008) with proportions of invariant sites, base frequencies, and the alpha parameter estimated from the data, was selected by the AICc in ProtTest v.2.4 (Abascal et al., 2005).

### PCR confirmation of disrupted genes

PCR was employed to confirm the sequences of the *dsrA, hupL*, and *hupS* genes. We included a second *Ca*. T. nelsonii metagenome sample, obtained from the same gastropod-attached community, in the confirmation screenings for the purposes of replication. PCR primers were designed using Primer3 (Untergasser et al., 2012). To capture most of the *dsrA* gene to include the intron sequence (bases 44-1320) the primers dsrA_TmargS10_F 5′-GAGTGGTCCTTGGCCTAGTT-3′ and dsrA_tmargS10_R 5′-GGGGACAGGGCTTTAGTCAT-3′ were used. Two primer sets were used to cover a portion of the *hupL* gene, the MITE sequence, the frameshift and the *hupS* gene. The primers S10_hupS-F 5′-ATGGATACAAACCGGTGCTT-3′ and S10-hupS-R 5′-GTCATGATGTTCCGCATAGC-3′ covered bases 1296…204 and the primers S10_hupSL-F 5′-ATGGATACAAACCGGTGCTT-3′ and S10_hupSL-R 5′-GTCATGATGTTCCGCATAGC-3′ covered based 1941…2624 in contig Ga0063879_1132. PCRs were performed using GoTaq^®;^ polymerase (Promega) with the addition of 10% dimethyl sulfoxide to stabilize hairpin structures.

### Nucleotide sequence accession numbers

The draft genome has been deposited with the National Center for Biotechnology Information, BioProject PRJNA266451 (*Candidatus* Thiomargarita nelsonii Hydrate Ridge). Raw sequence reads were deposited in the NCBI Short Read Archive. The binned Bud S10 genome was annotated and is publically available through the Joint Genome Institute's IMG website, Gold ID Ga0097846 (non-hybrid assembly) and Ga0063879 (hybrid assembly). All contigs greater than 1500 bps of the pre-binned metagenome hybrid assembly were annotated by IMG and are publically available through IMG/M, Gold analysis ID Ga0064232.

## Results

### Comparison to previous genome assembly and genome completeness

Prior to producing the hybrid assembly reported on here, an assembly using only the Illumina reads was performed with the metagenome assembler MetaVelvet (Namiki et al., 2012) followed by Mimimus 2 (Sommer et al., 2007). The assembly yielded 144,811 contigs (N50 = 2571), and the *Thiomargarita* bin derived from this initially assembly contained 2497 contigs greater than 2000 bps in size (12.97 MB). Subsequently, the same Illumina reads were co-assembled with PacBio CCS reads and then scaffolded with PacBio CLR reads. This hybrid metagenome assembly resulted in 3326 contigs of which 2370 contigs were greater than 1500 bps long and were used in the ESOM binning (Supplementary Material 1). The final *Ca*. Thiomargarita bin reported on here contains 439 contigs (7.71 MB) (N50 scaffold length = 36,326) with an average of 145-fold genome coverage. Only the contents of this *Ca*. Thiomargarita bin are discussed in the remainder of this report. Of the 7525 protein encoding genes, 56.71% had a predicted function.

The IMG pipeline assessed the binned genome as 86.53% complete. We further assessed genome completeness by examining the ribosomal polymerase, rRNA genes, tRNA, tRNA synthase genes and the ribosomal proteins. The binned genome contains *rpoA*, and two copies of *rpoB* (one co-located with *rpoC*) and one set of rRNA genes in a single operon. The contig containing this operon terminates in an incomplete 16S rRNA gene. The 23S rRNA gene is interrupted by three introns at positions 2038.2823, 2838.3111, and 3135.3851. Sister taxa “*Candidatus* Maribeggiatoa Orange Guaymas Basin” (hereafter referred to as BOGUAY) (MacGregor et al., 2013c) and “*Candidatus* Thiomargarita sp. NAM92” also possess introns in their 23S rRNA gene; however, neither have an intron in the third position (3135.3851). The resulting full-length 23S rRNA gene in Bud S10 is 5183 bases long. These introns did not match with introns in the Rfam database and further analyses regarding the phylogeny of the introns and their capacity to be self-catalytic was not determined.

A total of 46 tRNA genes are present and cover the 20 standard amino acids, except as in BOGUAY, tRNA-Arg-TCT and tRNA-Leu-TAA were missing from the annotated genes. MacGregor et al. (2013a) determined that they were present in BOGUAY but interrupted by putative group I introns. BLASTN analyses of these interrupted tRNAs in BOGUAY against the Bud S10 genome revealed similar interrupted tRNAs (tRNA-Arg-TCT contig Ga0063879_1007, bases 79335.79658) (tRNA-Leu-TAA contig Ga0063879_1068, bases 17090.17442). All tRNA synthetase genes were present except threonyl-tRNA synthetases and the glutminyl-tRNA was interrupted by two stop codons (Ga0063879_05998-6000). The binned genome also contains all of the ribosomal proteins with the exception of *rplT* (L20) and *rmpI* (L35). *rplD* (L30) was not annotated by the IMG pipeline, nor were we able to find it in the metagenome assemblies. L30 is a non-essential protein (Akanuma et al., 2012) but is found in most bacteria. However, the absence of L30 has been noted in candidate phyla with small genomes and self-splicing introns in their ribosomal rRNA (Brown et al., 2015), and in some host dependent strains, some cyanobacteria and the *Planctomycetes*–*Verrucomicrobia*–*Chlamydiae* superphylum (Lecompte et al., 2002; Yutin et al., 2012; Lagkouvardos et al., 2014).

### Major metabolic pathways

#### Carbon acquisition

The Bud S10 genome contains putative genes for the Calvin Benson Bassham-pathway with RuBisCO Form II (*cbbL*) performing the catalytic step of fixing CO_2_. Like the BOGUAY genome, Bud S10 does not possess genes encoding fructose 1,6-bisphosphatase or sedoheptulose 1,7-bisphosphatase. MacGregor et al. (2013a) postulated that these functions may be dependent on a pyrophosphate (PPi)-dependent 6-phosphofructokinase (*pfkA*) as it has been proposed for some gammaproteobacterial endosymbionts (Kleiner et al., 2012; MacGregor et al., 2013a). As in the BOGUAY genome, there were two putative genes encoding *pfkA* (Ga0063879_01082, Ga0063879_04320). The genome also contains the genes for a complete reverse TCA cycle including the three key genes 2-oxoglutarate ferredoxin oxidoreductase (*korAB*) (Ga0063879_04149-51), pyruvate oxidoreductase (*porABCD*) (Ga0063879_05883), and ATP citrate lyase (*aclAB*) (Ga0063879_05528-9). Bud S10 may not have a complete TCA cycle. The genome has a 2-oxoglutarate dehydrogenase, but a stop codon is present within the gene (Ga0063879_04055-6). Bud S10 also lacks a glyoxylate shunt as well as the genes for carboxysome synthesis. The lack of a viable 2-oxoglutarate dehydrogenase may be indicative of an obligate autotrophic lifestyle (Wood et al., 2004). However, Bud S10 possesses genes for ABC-type amino acid and peptide transporters, in addition to a dicarboxylate transporter which are suggestive of a mixotrophic lifestyle (Schulz and de Beer, 2002). The genetic potential for methylotrophy and alkane degradation (alkane 1-monoxygenase), common in hydrocarbon seep bacteria, was not observed in the genome.

#### Respiration, chemotaxic motility, and proton motive force

Only respiratory pathways involving O_2_ and inorganic nitrogen as terminal electron acceptors were detected in the genome. In Bud S10, aerobic respiration terminates with a cbb3-type cytochrome, which has a high affinity for O_2_ and is known to be specific to strains adapted to microaerophilic environments (Molinas et al., 2011). A catalase, a key enzyme for handling oxidative stress, was not found, nor did we find it in other marine *Beggiatoaceae*. However, Bud S10 possesses an operon of other genes for reducing oxidative stress including superoxide dismutase, desulfoferrodoxin, and cytochrome c peroxidase. *Thiomargarita* spp. are not known to be motile via flagella or gliding motility; however a rolling movement has been observed in some ecotypes (Salman et al., 2011). The genome of Bud S10 contains genes associated with twitching motility (*pilGHIJTU*) as well as those associated with a chemotactic response to a stimulus such as O_2_ (*cheABRWY* and a methyl-accepting chemotaxic protein).

The genome of Bud S10 has the complete Complex I for oxidative phosphorylation (NADH subunits A-N), two operons encoding succinate dehydrogenases, and two operons encoding F-type ATPases. The genes encoding a vacuolar type H^+^ translocating ATPase (ntpABCDEFIK) are also present. A V-ATPase has been shown to be responsible for generating a proton-motive force across the membrane of a marine *Beggiatoa*'s nitrate vacuole resulting in intra-vacuolar acidity (Beutler et al., 2012). Additionally, the genome of Bud S10 had two copies of the genes of the rnf complex (*rnfABCDEFG*). The rnf complex is a Na^+^-pumping ferredoxin:NAD^+^ oxidoreductase that generates a chemiosmotic gradient of Na^+^ for ATP production.

Analysis of the Bud S10 dataset revealed two complete pathways for the dissimilatory reduction of nitrate. ${\text{NO}}_{3}^{-}$ may be reduced to ${\text{NO}}_{2}^{-}$ in the periplasm via a periplasmic nitrate reductase (*napABCDFGH*) and via a membrane bound nitrate reductase (*narGHIJ*), for which there are two complete operons, each containing a putative cytochrome *c*-like gene. Nitrite may then either be reduced to ${\text{NH}}_{4}^{+}$ via a *nirBD*-type nitrate reductase or to N_2_ via a *nirS*-type nitrite reductase followed by a nitric oxide reductase (*norBCDQ*), and finally a nitrous oxide reductase (*nosZ*) (${\text{NO}}_{2}^{-}$ > NO > N_2_O > N_2_). The octaheme cytochrome c reductase (BOGUAY_0691) that was shown to have nitrite reductase, hydroxylamine oxidase, and hydrazine oxidase activities in the BOGUAY (MacGregor et al., 2013a,b), was identified in the genome of Bud S10 (Ga0063879_0044), as were other multi-heme cytochromes of unknown function (Ga0063879_04661 and Ga0063879_05965). Several inorganic nitrogen transporters were also identified: a nitrate:nitrite antiporter (*narK*) (Ga0063879_02276), a formate/nitrite transporter (Ga0063879_00790) and two ammonia transporters (Ga0063879_04108, Ga0063879_04110).

#### Lithotrophy

**Figure 2:**
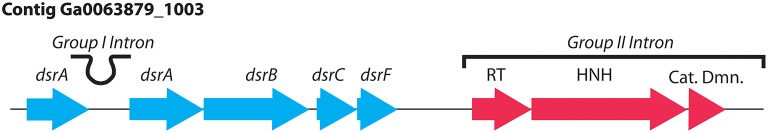
**Oxidative dissimilatory sulfite reductase operon with a group I intron interrupting the ***dsrA*** gene and a group II intron containing accessory genes that facilitate the intron's insertion into the genome**.

**Figure 3:**
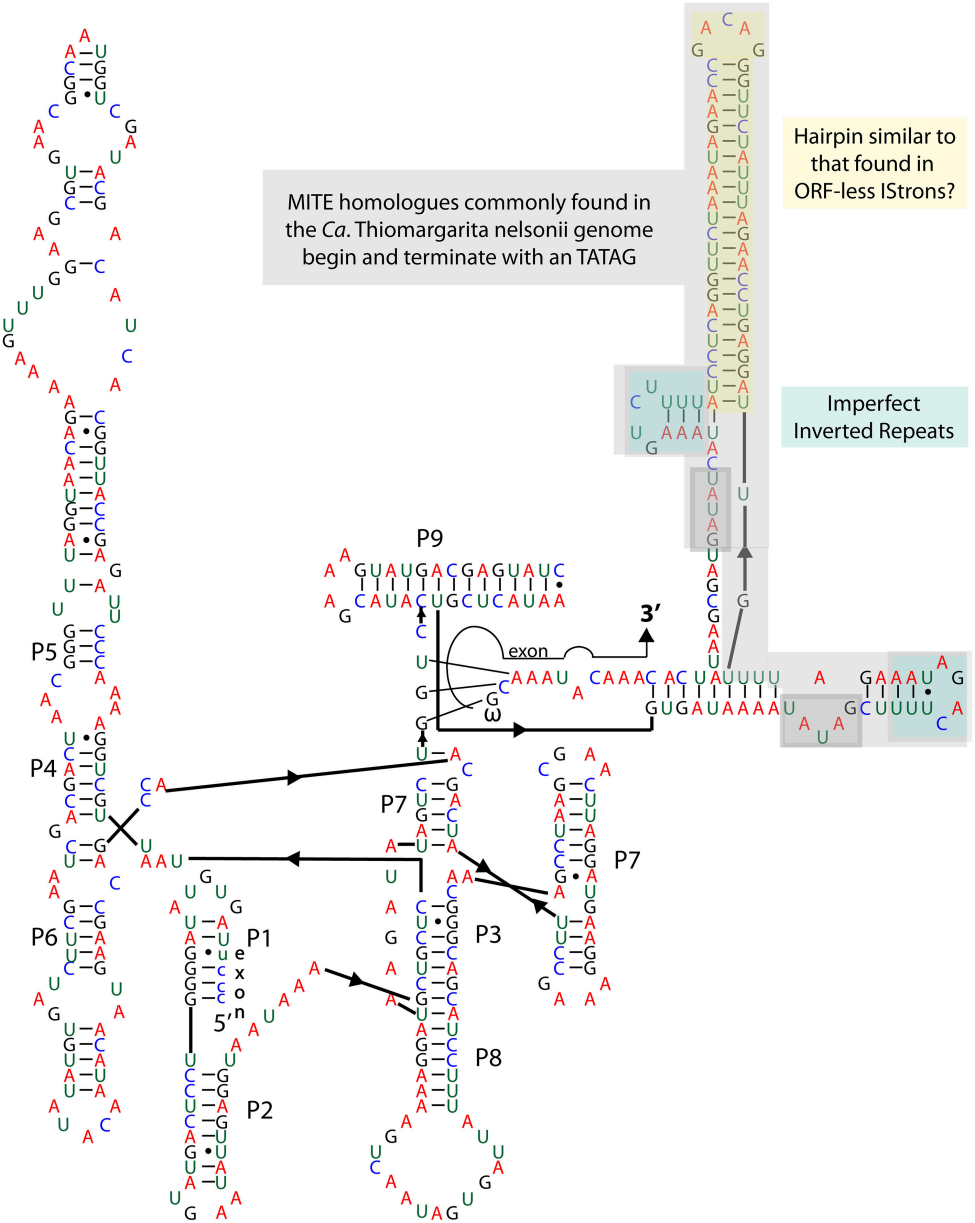
**Group IA2 intron in the oxidative dissimilatory sulfite reductase gene ***dsrA*****.

The genome of Bud S10 indicated that this strain could utilize a number of reduced inorganic sulfur substrates, arsenite (${\text{AsO}}_{3}^{-}$) and potentially H_2_ as electron donors. The sulfide oxidation pathways, operon structure and gene copy numbers were mostly consistent with vacuolated *Beggiatoaceae*. The oxidation of H_2_S could occur via a sulfide:quinone oxidoreductase, and/or flavocytochrome c, present as one and two gene copies respectively. Thiosulfate oxidation could occur via the sox system (*soxABXYZ*) co-located on an operon with *qmoABC*. Elemental sulfur (cyclooctasulfur), an intermediate of sulfide oxidation, could be oxidized to ${\text{SO}}_{3}^{2-}$ via the complete oxidative dissimilatory sulfite reductase system (*dsr*ABCEFHKJLMOP). There is a group II intron that is downstream of *dsrABEF*, which neighbors on the opposite strand *dsrL*, the gene for a sulfur relay protein, Figure 2. The other *dsr* genes are on another contig. Another distinctive feature is that *dsrA* (Ga0063879_66054-5) was found to be interrupted by a group I intron (see analysis below, Figures 2, 3). In addition to cyclooctasulfur, polysulfides were recently identified as intermediates in sulfide oxidation in *Beggiatoa* (Berg et al., 2014). Bud S10 may utilize the polysulfides oxidatively or perhaps reductively via a polysulfide reductase-like operon (*phsABC*) (Eddie and Hanson, 2013; Weissgerber et al., 2013). Bud S10 had the potential to further reduce ${\text{SO}}_{3}^{2-}$, either via a sulfite dehydrogenase (*sorA*) or an adenylylsulfate reductase (*aprAB*) and sulfate adenylyltransferase (*sat*). Other operons of unknown function contain genes for putative lithotrophic growth on reduced sulfur. For example, an operon Ga0063879_02957–Ga0063879_02962 contains a gene similar to the polysulfide reductase membrane anchoring protein (*phsC*), along with a number of cytochromes, an ATPase and hypothetical proteins. A number of operons also contain genes for rhodanese-like enzymes which are thought to play a role in lithotrophic growth on certain sulfur species (Weissgerber et al., 2013).

*Thiomargarita* spp. have not been previously shown to utilize hydrogen as an electron donor. However, Bud S10 appears to possess genes for a Ni-Fe hydrogenase (*hupL* Ga0063879_05378-9; *hupS* Ga0063879_05380) and some hydrogenase accessory proteins *hypABCEF* on a separate contig (Ga0063879_1018). Additionally, 19 putative genes (mostly hypothetical genes) separated *hypE* from the cluster of other accessory genes. However, *hupL* was interrupted by a miniature inverted-repeat transposable element (MITE) (base positions 1357.1561(-), see below and Supplementary Material 2). Regardless of the MITE, neither *hupS*, nor *hupL*, appear to be complete genes or they may be fused. This disruption or gene fusion occurred upstream of the MITE sequence. PCR products of the *hupSL* region of the whole-genome-amplified DNA of both Bud S10 and an additional single cell sample were consistent with the assembled genome. No additional PCR products were detected, thus it appears there are not multiple versions of the region, which could occur in a polyploid organism. A BLASTX analysis against GenBank of the *hupS* and *hupL* sequences with the MITE and frameshifts removed indicated that the likely class of the *hupSL* is the newly described group 2c hydrogenase (top BLASTX score for both genes was *Methylobacter tundripaludum, hupS* WP_031435956 *e*-value 3e^−79^, 59% identity, *hupL*, WP_027150633, *e*-value 9e^−100^, 49% identity) (Greening et al., 2015). We examined the *hupL* sequence for the two defining metal ligating cysteine residues, L1 and L2 in NiFe hydrogenases (Vignais and Billoud, 2007; Greening et al., 2015). The L1 motif is absent but the L2 motif is most consistent with a group 2c hydrogenase (consensus group 2c: SFDxCLVCTVH; *Thiomargarita*: SHDaCLVCTVH). Group 2 hydrogenases are cytosolic. Group 2a hydrogenases in the Cyanobacteria provide electrons under aerobic conditions and recycle H_2_ generated by cellular processes, while group 2b are thought to be hydrogen sensors and thus, perform a role in cellular regulation. Group 2c hydrogenases were, until recently, classified as group 2a. However, group 2c hydrogenases have distinct L1 and L2 motifs and may be co-transcribed with diguanylate cyclases/phosphodiesterases, which are proposed to regulate global transcription based on fluctuating H_2_ conditions (Greening et al., 2015). These hydrogenases are uncommon and mostly restricted to aquatic sulfate-reducing bacteria and methylotrophic bacteria. Since *Thiomargarita*'s *hupSL* genes are incomplete perhaps by deletion, gene rearrangement or fusion, we cannot perform full phylogenetic or structural analyses, nor can we confirm that a diguanylate cyclase or a phosphodiersterase may be co-transcribed with these genes since *hupSL* are at the terminal end of a contig.

**Figure 4:**
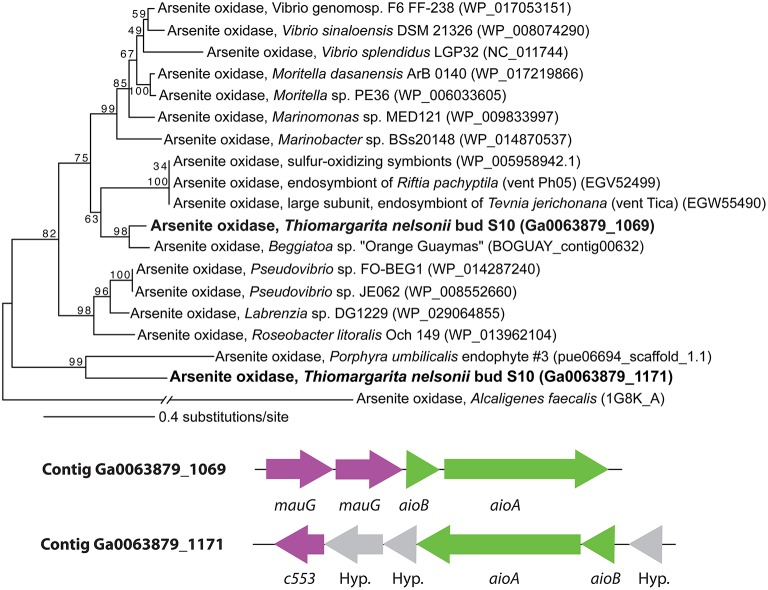
**The arsenite oxidoreductase operons and a maximum likelihood tree of arsenite oxidoreductase subunit ***aioA*****. Numbers indicate bootstrap support for each node.

The genome of Bud S10 also contained two putative arsenite oxidoreductases (*aioBA*) in truncated operons at the terminal ends of contigs, Figure 4. One set of *aioBA* genes (Ga0063879_03942–03943) was preceded by two cytochrome c peroxidases (*mauG*) in an operon, an arrangement that is similar to those found in other marine Alpha- and Gammaproteobacteria (Li et al., 2013). The other set of *aioBA* genes (Ga0063879_05976–05977) were distinctly different (Figure 4, Supplementary Material 3). The large subunit *aioA* contained a large insert that we have not found in other known *aioA* genes; however, this region lacked direct or inverted repetitive elements indicative of a mobile element. Furthermore, a BLASTN against this 379-base region against Bud S10's genome, the Rfam database and NCBI's CDD database produced no hits. The terminal end of the contig would be the expected region for the *mauG-mauG* gene set. However, we found no homology with *mauG*, but rather repetitive and palindromic regions discussed below. It is nearly universal to find co-occurring arsenic resistance genes, phosphate stress response genes and the *aio* genes in “arsenic islands” (Li et al., 2013). However, both of the Bud S10 *aioBA*-containing contigs were short contigs and neither contained the other genes typically found in arsenic islands. Instead, both contigs contained additional cytochromes. However, genomic positioning may suggest a role in stress response since downstream from one *aioBA* was a universal stress protein gene (*uspA*), and downstream from the other, a carbon starvation protein (*cstA*).

#### Potential phosphorus and carbon sequestration

In both bacteria and eukaryotes, polyphosphate can be stored in membrane-bound vacuoles, called acidocalcisomes. Polyphosphate inclusions were observed in *Thiomargarita namibiensis* and the hydrolysis of these polyphosphate granules has been linked to phosphorite formation in pore waters inhabited by dense populations of *Thiomargarita* spp. (Schulz and Schulz, 2005). Typically, acidocalcisomes are acidic and contain high levels of divalent cations, most notably calcium and magnesium (Docampo et al., 2005; Seufferheld et al., 2008; Forbes et al., 2009; Rao et al., 2009). The genetics of acidocalcisomes in bacteria and, to lesser extent eukaryotes, is poorly understood. The Bud S10 genome possessed a number of genes for inorganic phosphorus (P_i_) acquisition including an ABC-type ATP-binding P_i_ transporter (*pstAB*), a P_i_ selective porin, a low-affinity P_i_ transporter (*PiT*), and a Na^+^/P_i_ co-transporter. Potential storage of P_i_ in poly-P granules occurs via a polyphosphate kinase I (*ppk1*). Other enzymes connected to poly-P metabolism in the genome included a broad specificity polyphosphate kinase 2 (*ppk2*), an exopolyphosphatase [*surE* but not *Ppx-GppA* (either PFAM 02541 or 02833)], (p)ppGpp synthase/hydrolase and poly-P glucokinase (*ppgK*). Bud S10 possessed several candidate divalent cation transporters, including a K^+^ dependent Na^+^/Ca^2+^ exchanger and a potential calcium-translocating P-type ATPase, which could contribute to divalent cation accumulation in the acidocalcisomes. A V-type ATPase could be involved in the generation of an acidic polyphosphate compartment. However, a recent study of a marine *Beggiatoa* strain that lacked a nitrate vacuole had unusual membrane bound polyphosphate granules that contained high levels of Ca^2+^ and Mg^2+^ but were not acidic (Brock et al., 2012). Thus, the V-ATPase may be important for other acidic compartments, e.g., the nitrate vacuole vs. polyphosphate granules. Polyphosphate-accumulating bacteria often store organic carbon granules such as glycogen and poly(R)-hydroxyalkanoic acids. Bud S10 could potentially store glycogen synthesized from ADP-glucose via a starch synthase (*glgA*), but not poly(R)-hydroxyalkanoic acids. These genetic findings are consistent with microscopic observations of *Thiomargarita namibiensis* that stained dark brown with iodine indicating the presence of a glucose-containing polymer (Schulz and Schulz, 2005).

### Assessment of mobile and/or repetitive elements

In general, our examination of the genome of Bud S10 revealed a number of characteristics that are atypical for bacteria. For example, 18% of the genome was intergenic. Forty two percent of the genes were classified as hypothetical, and introns and/or MITES disrupted numerous ribosomal RNA and protein-encoding genes. Additionally, some of the most abundant gene motifs detected here indicate a complex developmental life cycle and/or genome plasticity. For example, one of the most common motifs detected in the annotation pipeline were genes for metacaspases (*n* = 59) (PFAM00656). In eukaryotes, caspases carry out programed cell death. Metacaspases are uncommon in bacteria (< 18%) and their roles are in general, poorly-understood (Asplund-Samuelsson et al., 2012). In Cyanobacteria they are correlated with complex life styles, e.g., multi-cellularity, filamentation, and dimorphism, and have been shown to be restricted to strains that do not possess streamlined genomes (Asplund-Samuelsson et al., 2012) including a filamentous, multicellular endosymbiotic strain that appears to undergo programmed cell death (Zheng et al., 2013). The number of metacaspases in the Bud S10 genome far exceeds those found in other sequenced genomes. Besides the Cyanobacteria, they occur in higher numbers in some symbiotic rhizobia, symbiotic methylotrophs, and *Myxococcus* species. While the highest number (*n* = 28) and the most diverse metacaspases were reported in a Bacteriodetes, *Haliscomenobacter hydrossis* DSM 1100, which has both single cell and filamentous chains that can exhibit branching morphology.

**Figure 5:**
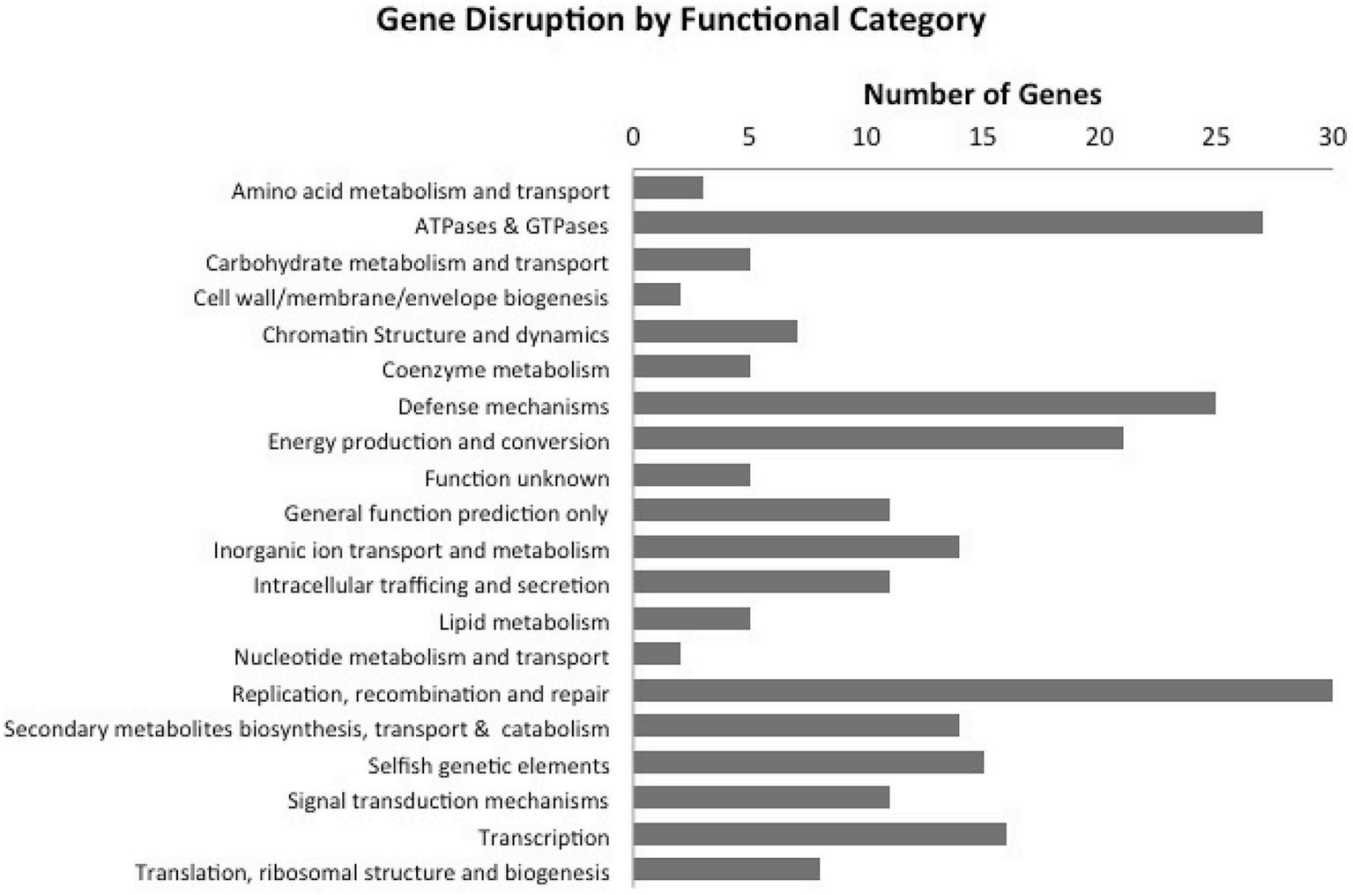
**Number of putatively disrupted genes with predicted function by functional category**. A total of 1120 out of 7125 genes predicted by GenBank were putatively disrupted and of those only 248 had a predicted function.

Other common motifs in the genome of Bud S10 were reverse transcriptases (*n* = 62) (PFAM00078), HNH endonucleases (*n* = 60) (PFAM01844) and PIN domain-containing genes (*n* = 64) (PFAM01850). PIN-domain-containing proteins are Mg^2+^ dependent single stranded ribonucleases (Clissold and Ponting, 2000). PIN domain containing proteins have been shown to inhibit transcription (Winther and Gerdes, 2011) and cleave mRNA by recognizing a hairpin in the RNA secondary structure (McKenzie et al., 2012a,b). A few of the other common PFAM motifs were indicative of mobile genetic elements such as group II introns and several types of transposons. Moreover, the GenBank annotation pipeline indicated that 1120 out of 7125 genes are disrupted. Some portion of the genes are likely disrupted by frameshifts or stop codons, whether real or via sequencing errors, and some putatively-disrupted genes are perhaps incorrectly annotated as such. However, we observed some trends in those genes in which GenBank predicted a gene function (Figure 5, Supplementary Material 4). Most notably, an abundance of ATPases of unknown function that typically contained P-loop motifs and AAA domains. The AAA domain (PFAM13304) was the most common motif in Bud S10 genome with 158 occurrences. The functions of AAA domain ATPases are diverse but include many core functions, including DNA replication, recombination, and repair; transcription; and protein folding (Iyer et al., 2004; Snider et al., 2008). We also observed that many of the disrupted genes were polymerases, transcriptional regulators and transporters. Therefore, we performed a brief assessment of Bud S10's genome stability or plasticity by examining mobile and/or repetitive elements in the genome. In addition to IMG annotation, RepeatModeler was employed to aid in identifying repetitive and palindromic sequences.

#### RepeatModeler detected repetitive motifs

RepeatModeler identified eight repetitive sequences that occurred greater than 15 times in the genome (ranging from 15 to 55 occurrences) and generated a consensus sequence for each (Supplementary Material 5). None of the consensus sequences contained motifs or homologs to the *dsrA* or rRNA gene introns, the *hupL* MITE, or families and motifs in the Rfam database. The consensus sequences were typically palindromic and a BLASTN of each against Bud S10's genome indicated that motifs in most of the consensus sequences occurred broadly across the genome (Supplementary Material 6). Some consensus sequences were group II introns, while others were DDE domain transposons, CRISPR arrays, and IS605 insertion elements, and their remnants as discussed further below. However, the identity and/or function of the other consensus sequences could not be determined. A detailed examination of the location of these sequences in relationship to encoding genes would facilitate greater understanding of these repetitive sequences, but such an analysis would be complicated by the large number of contigs and hypothetical genes, and therefore lies beyond the scope of the analysis reported on here. Recently, a heptamer sequence, TAACTGA, was found to occur as direct and indirect repeats in other marine *Beggiatoaceae* and was proposed to play a role in transcript regulation (MacGregor, 2015). We found 1175 occurrences of this sequence, in some cases as direct repeats, in Bud S10's genome; however, we did not find TAACTGA in any of the Repeat Modeler consensus sequences.

#### Group I introns

Group I introns are usually, but not always, self-splicing mobile genetic elements that excise from the flanking exons once transcribed into RNA (Nielsen and Johansen, 2009; Hausner et al., 2014). As noted above, *dsrA* is a key gene in the sulfur oxidation pathway of vacuolate sulfur-oxidizing bacteria, and we anticipated its presence in the genome of Bud S10. However, *dsrA* appears to be interrupted by a group I intron (Figure 3). This finding led us to confirm the presence of the intron and determine if other versions of the gene were present by PCR. Amplification of the *dsrA* gene from Bud S10, as well as from a second single-cell amplified genome from the same *Thiomargarita* population, produced a single PCR product, and subsequent Sanger sequencing confirmed the presence of the putative intron. A search for homologs in the Rfam database yielded only one motif hit, a group I intron, *e*-value 5.4e^−13^, bit score 298. One key feature of most group I introns is a GU pair at the 5′ splice site. The other is the catalytic domain in the P7 Region where an exogenous guanosine binds and initiates the cleavage of the splice site. A BLASTN of the putative *dsrA* intron against the NCBI database revealed sequence homology with a group IA2 intron found in protein-coding genes of unknown function (Orf142) in the *Staphylococcus* bacteriophage, “Twort.” Homology was indicated for the P7 region, as well as for the P3, P8, J6/7, and J3/4 regions (intron orf142-I2, accession number 2RKJ_C, *e*-value 0.011, 60 of 79 identities) (Landthaler and Shub, 1999; Paukstelis et al., 2008). The *dsrA* intron also shares sequence homology with the P4, P5, and partial P6 domain of another intron in a ribonucleotide reductase within the Twort genome (intron nrdE-I2, accession number AF485080 *e*-value 9e^−10^, 84 of 111 identities) (Landthaler and Shub, 1999; Landthaler et al., 2002). Sequence and structural similarities in the Twort introns suggest common origins (Landthaler et al., 2002). Within Twort's nrdE-I2's P6 domain is a loop containing a DNA-nicking endonuclease. This feature is absent in the *dsrA* intron. Additionally, the P9 region containing a large (51 bp) hairpin in the *dsrA* intron is not found in the Twort introns. Both the 5′ and 3′ splice sites, as well as the catalytic regions in the *dsrA* intron, are consistent with other group IA2 introns, thus the *dsrA* intron appears to be a self-splicing ribozyme.

A BLASTN analysis of the *dsrA* intron indicated that the introns in the 23S rRNA gene and the tRNA genes introns were not close homologs. However, there were more than 100 distinct contigs in the genome that contain a portion of the *dsrA* intron that includes the long hairpin in the P9 region (Figure 3), but not the regions with sequence similarity with Twort's introns. Most commonly, homologs to the region of the sequence coding for the large P9 hairpin are conserved across the genome, predominantly in intergenic regions. But not uncommonly, a larger section of the *dsrA* intron is also found with the hairpin and these homologs terminate on both ends with the direct repeat TATAG (Figure 3). Within this section there is also a one base mismatch inverted repeat sequence. This base arrangement (DR-IR-xx-IR-DR) is a common feature of mobile elements including insertion sequence (IS) elements and MITES. The TATA at the terminal ends of the sequence is a very common feature of a MITE but it is also found in the IS630 family from which they are thought to be derived. If this region constitutes a distinct mobile element, independent of the intron elsewhere in the genome, the lack of a protein-encoding region that would promote its excision (as would occur in a IS element) indicates that it is more likely a MITE. Although a molecular symbiosis between a MITE and a group I intron has not been previously reported, a similar symbiosis has been observed between an IS element and an intron, which is referred to as an IStron (Braun et al., 2000). Interestingly, the long hairpin in the P9 region is reminiscent of a motif found in IStrons. However, in these mobile elements, a transposase is located within the loop of the large hairpin. A recent genome survey found that IStrons were restricted to a few members of the Firmicutes and Fusobacteria (Tourasse et al., 2014). Unlike group I introns, IStrons invade many types of protein-coding genes and are often found without some, or all, of the IS components, making them appear more like a MITE.

#### Group II introns

Group II introns are found in bacteria and eukaryotic organelles, and are thought to be the precursors to eukaryotic spliceosomal introns and retrotransposons (Lambowitz and Zimmerly, 2011). They fold into a structure with six domains upon transcription. Many carry a reverse transcriptase and an endonuclease to promote their mobility and integration into the host genome, as well as a maturase within the fourth domain that assists in self-splicing. Some group II introns of the IIB1 family have some distinct alterations from other group II introns to include a LAGLIDADG family homing endonuclease. This feature is present in the group II intron found in *Thiomargarita* 16S genes (Salman et al., 2012). The IMG/ER annotation pipeline identified 57 group II introns, all of which were of the IIB2-type. RepeatModeler identified 55 sequences of these introns aligning to a consensus sequence that encode the 5′ RNA encoding region, a reverse transcriptase, a maturase, a HNH endonuclease and a group II catalytic region. A BLASTN of the consensus sequence revealed elements of these introns on 100 contigs in the genome assembly indicating that remnants and non-protein encoding versions of this intron occur in the genome as well. Many of these group II introns neighbored key genes in cellular replication and repair. Examples include genes for DNA helicase, ribosomal proteins L7, S14, DNA primase, ribosomal recycling factor protein, and putative AAA domain ATPases. In other cases, the group II introns interrupt operons key to *Thiomargarita* spp. metabolism such as the periplasmic nitrate reductase. In other cases they flank operons containing genes for dissimilatory nitrite reductase, dissimilatory sulfite reductase (Figure 2), and the nitrous oxide reductase.

**Figure 6:**
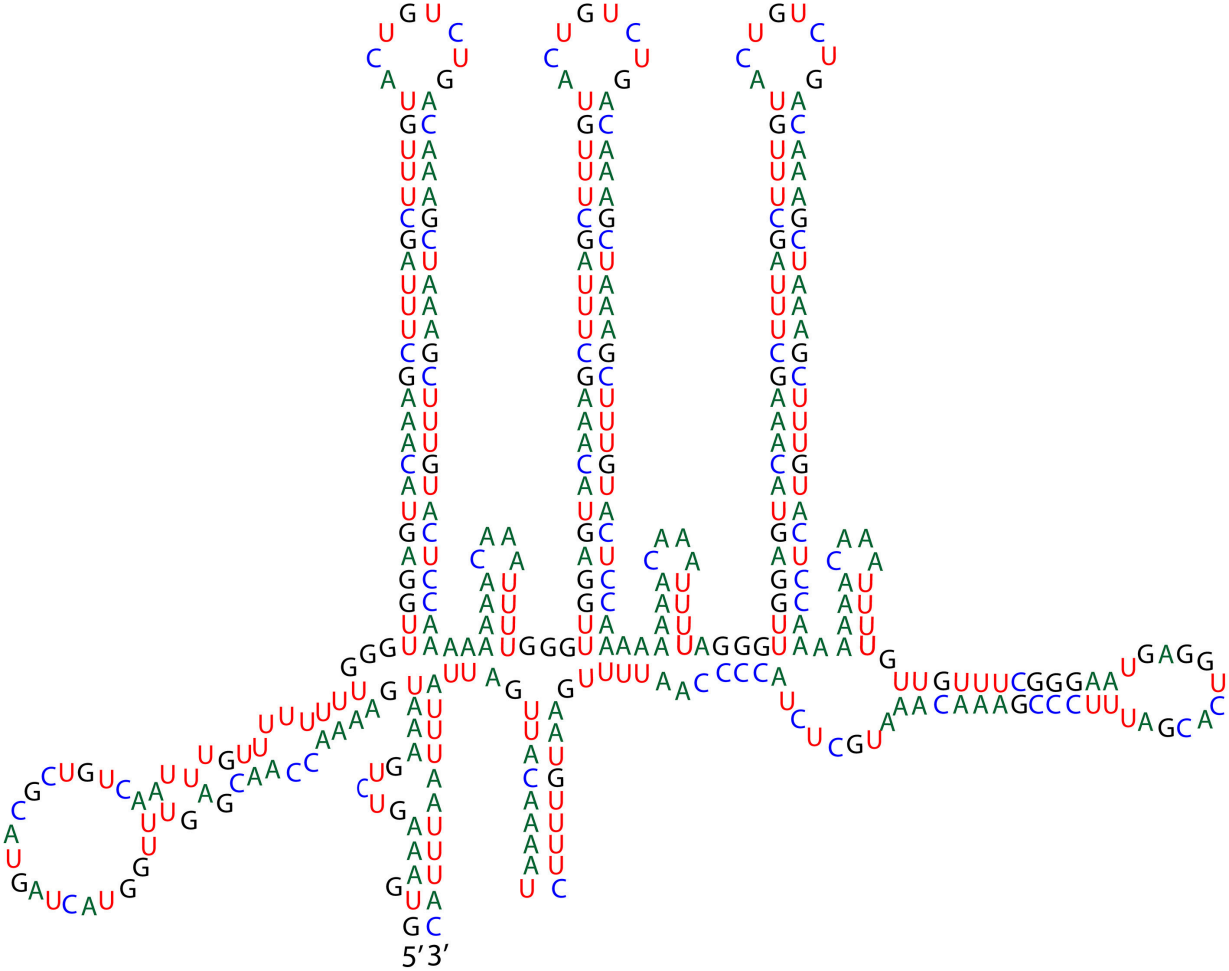
**Example of a repetitive element identified by RepeatModeler**.

RepeatModeler also identified a family of repetitive and palindromic sequences that, in one case, precedes a gene containing group II-like reverse transcriptase, homing endonuclease, and maturase motifs, but that fell below cutoff scoring for a group II intron. This hypothetical gene (Ga0063879_05465) was one of the top scoring BLASTN hits of the Repeat Modeler consensus sequence (“Family 24,” BLASTN *e*-value 4e^−45^). This sequence does not have motifs found in group II introns but it appears to encode RNA (Figure 6). Within the same 16,748 bp contig as Ga0063879_05465 (Ga0063879_1138), there were three additional BLASTN hits with the same consensus sequence, two of which were in intergenic space and the third at the terminal end of the contig. Indeed, a BLASTN analysis indicated that homologs of the consensus sequence occur broadly across the genome. One gene upstream of these pseudo-group II intron genes were two putative restriction endonucleases and two BLASTN hits to a different RepeatModeler consensus sequence (“Family 176,” *e*-value 5e^−23^; 4e^−11^). We found nothing similar to either consensus sequences in our searches of gene and motif databases. But the orientation of Families 24 and 176 to the group II-like protein encoding gene components is suggestive that families 24 and 176 may encode intron-like RNAs.

#### Insertion sequence elements/transposases

IS elements are autonomous mobile elements that are common in the genomes of bacteria. They have a characteristic structure (DR-IR-transposase-IR-DR) and a promoter for transcription of the transposase in the 5′ inverted repeat (Siguier et al., 2014). There are many families of IS elements and they are primarily classified based on the type of transposase they contain. The IMG/ER pipeline identified 32 IS605-like IS elements (TIGR01766). In addition, the IMG pipeline identified 13 putative IS200-like IS elements, (IPR002686). The IS200/IS605 family of IS elements are unique among known IS elements in that they encode a HUH endonuclease that recognizes a terminal imperfect palindrome and then transposes it into a single strand of genomic DNA (He et al., 2013). They are also unique in that they have been found in IStrons (see Tourasse et al., 2014, and references therein). This IS family also has the potential to transform or decay into a unique MITE-like structures called palindrome-associated transition elements (PATEs) (Dyall-Smith et al., 2011; Siguier et al., 2014). PATEs have been observed in archaea, some cyanobacteria and *Salmonella*. Furthermore, this IS group has been shown to be domesticated by the host to modulate gene expression in a few enterobacteria [bacterial interspersed mosaic elements (BIMES)]. Because RepeatFinder detected the remnants of IS605-like IS elements in the genome of Bud S10, we predict that the genome of also contains MITE-like features derived from these groups of IS elements.

The IMG annotation pipeline also identified 55 other putative transposases/IS elements, either containing the classic DDE transposase domain found in IS elements (Siguier et al., 2014) or a PD-(D/E)XK homing endonuclease domain. Of the DDE domain-containing genes, five are ISXO2-like transposases (PFAM12762), two are in the IS4/5 family (PFAM13340), and 20 other putative transposases contain DDE domains (PFAM13612, PFAM01609, PFAM07592, PFAM13358, PFAM13737, PFAM13586). The remaining 28 putative transposases/invertases (TIGR01784) contain a PD-(D/E)XK_2 homing endonuclease domain (PFAM12784). Many of these genes fall below the trusted bit score and include putative Yhg-A transposases that contain homologs to the *hupL* MITE. PD-(D/E)XK endonucleases have a broad range of functions and include *xisH* discussed below (Zhao et al., 2007). The IS elements that contain PD-(D/E)XK endonucleases are poorly understood.

#### MITEs

MITEs are small transposable elements that typically encode a recognition sequence, but not a transposase, and are thought to be derived from IS elements (Delihas, 2011; Darmon and Leach, 2014). The mobility of MITEs are likely to be via recognition and excision by endonucleases, e.g., the parental IS element encoded elsewhere in the genome (Bardaji et al., 2011). MITEs are common in eukaryotic genomes, but are less well studied in bacteria even though they appear to have significant impacts on the host. They have been found to carry open reading frames (ORFs), inactivate genes via disruption, affect transcription, create gene fusions, and cause large deletions and gene rearrangements (see Darmon and Leach, 2014, and the references therein). As previously mentioned, a MITE-like sequence disrupts the hydrogenase subunit *hupL* in Bud S10. The *hupL* MITE is a typical MITE sequence. The sequence is capped on either end by a 10 bp direct repeat. Just internal to the direct repeats is a 19 bp inverted repeat (Supplementary Material 2). A single bp is shared by the direct and inverted repeats. We did not find a likely candidate for potential parental IS element family to this MITE in the ISfinder database. A BLASTN of the *hupL* MITE against Bud S10's genome resulted in hits on 260/439 contigs. One of the tops hits (*e*-value 3e^−83^) is located within a putative Yhg-A transposase (Ga0063879_06094-06095) and immediately precedes the sulfide:quinone oxidoreductase in the ORF. MITEs and MITE-like structures are known to be derived from IS elements that have DDE domain endonucleases and HUH endonucleases, not a PD-(D/E)XK_2 endonuclease, as in putative Yhg-A transposases. Thus, the *hupL* family of MITEs may be the first MITEs detected that originated from a PD-(D/E)XK_2 endonuclease containing IS element.

The MITE found in the *dsrA* group I intron, and across the genome, is unrelated to the *hupL* MITE. The parental origin of MITEs can be difficult to determine, particularly if the IS element is no longer within the genome, and we did not find a strong candidate IS element family in the ISfinder database. MITEs commonly possess TATA at the terminal ends and they can fold into large hairpin structures as in Bud S10. We found these MITEs sequences most similar in structure to MITEs like Correia elements (Delihas, 2008; Siddique et al., 2011). Correia elements have been shown to encode promoters for transcription. The TATA motifs function as Pribnow box promoters (TATA-box promoter in Eukaryotes). A second box promoter region exists in the inverted repeat region. Correia elements can affect transcription both from the 5′ and 3′ direction. The sequences internal the TATA-box promoters are significantly divergent from that of the Bud S10 MITE. Thus, the function(s) of these MITEs would need to be experimentally determined.

#### Inteins

Inteins are mobile elements that encode a peptide that splices out from a host protein after translation leaving a functional host protein. The IMG annotation pipeline identified four genes putatively containing an intein domain. As in BOGUAY, inteins were found in a *dnaB* replicative DNA helicase (Ga0063879_01777) and in a *dnaE* DNA polymerase III (Ga0063879_00371). Inteins were also identified in two ribonucleoside-diphosphate reductases- (RNR): one in an aerobic reductase subunit A (TIGR02506) (Ga0063879_01956) and the other in an anaerobic reductase (TIGR02487) (Ga0063879_04057). With the exception of the *dnaE* intein, all inteins contained either a LAGLIDADG-like domain or a homing endonuclease domain, which likely permitted the initial insertion of the intein into the genome. The genes that contain inteins work in concert for DNA replication. Ribonucleoside-diphosphate reductases convert NDPs to dNDPs and their activity is tightly regulated for replication and repair (Herrick and Sclavi, 2007). *dnaB* unwinds the dsDNA and *dnaE* adds the dNDPs generated by the RNR to the ssDNA template. It is not uncommon to find inteins in genes involved with DNA replication (Darmon and Leach, 2014). However, a review of the PFAM database (Finn et al., 2013) showed that the number of bacterial strains with four or more intein domains, as was observed here, (PFAM14890) is very uncommon (*n* = 3).

#### fdxN excision elements

Heterocyst formation in the cyanobacteria, which is a type of cell differentiation, occurs via DNA rearrangements in the genes *hupL*, a nitrogenase gene (*nifD*) and a heterocyst specific ferredoxin (*fdxH*) (Kumar et al., 2010). These rearrangements are performed by site-specific recombinases, of which *xisA* (*hupL*) and *xisC* (*nifJ*) are of the tyrosine or phage recombinase family (Nunes-Düby et al., 1998) and *xisF* is of the large serine recombinase (resolvase or IS605-like) family (Smith and Thorpe, 2002). Serine recombinases often require helper genes. Recently, it was demonstrated that the pair of helper genes, *xisHI*, for *xisF* are likely an endonuclease and a recombination directionality factor, respectively (Hwang et al., 2014). *Beggiatoa* and a few other filamentous or pleomorphic strains have also been shown to possess *xisHI* but not *xisF* (MacGregor et al., 2013c). Bud S10's genome possesses *xisHI* genes: four *xisH* (PFAM08814) and seven *XisI* (PFAM08869). The genome also had eight putative site-specific serine type recombinases (COG2452), many of which are near transposons but none of which appear to be homologs of *xisF*.

## Discussion

The genome of Bud S10 revealed a *Candidatus* Thiomargarita nelsonii phylotype that has the genetic potential to oxidize a large variety of sulfur species, hydrogen, and arsenite using oxygen or nitrate as terminal electron acceptors. However, the discovery of a very high number of mobile genetic elements in the draft genome, including some that interrupt genes in the sulfur and hydrogen oxidation pathways, complicates the interpretation of the functionality of some of these pathways. The degree of plasticity seen in the Bud S10 genome is exceedingly rare in bacteria and archaea. In general, a high degree of genome instability is typically seen in host-associated strains (but not ancient symbioses), some Cyanobacteria, and in certain extremophiles (Darmon and Leach, 2014). Genome instability may be a precursor to genome reduction by inactivating non-essential genes, promoting an obligate host-association (Siguier et al., 2014). We hypothesized that polyploidy in *Thiomargarita* spp., (Lane and Martin, 2010) might reduce the deleterious effects of the insertion of certain mobile elements, since multiple versions of the genome may exist in a single cell. However, we did not observe alternative versions of the *dsrA* and *hupSL* genes in the metagenome, nor were we successful in our attempts to produce multiple PCR products for these genes from both Bud S10 or another single *Thiomargarita* cell recovered from the same provannid gastropod. Therefore, we hypothesize that other factors in addition to polyploidy may result in the apparent genome plasticity observed in the Bud S10 genome.

In general, the increased rate of gene rearrangements and protein variants promoted by mobile genetic elements may increase the adaptability of an organism to new and/or extreme habitats (Lin et al., 2011; Darmon and Leach, 2014). Furthermore, mobile elements, as well as short repetitive elements similar to the heptamer recently discovered in the *Beggiatoaceae* (and a few Cyanobacteria and Bacteriodetes), have been demonstrated to promote phase and antigenic variation (see Darmon and Leach, 2014; MacGregor, 2015 and references therein). For example, excision of mobile elements can function as “on” and “off” switches to disrupted genes. Phase variation and antigenic variation have been demonstrated to play roles in numerous processes such as capsule formation, adhesion, nutrient acquisition, biofilm formation and host interactions, e.g., invasion and pathogenicity. *Thiomargarita* spp. requires access to both sulfide and oxygen (or nitrate), substrates that co-occur only in diffusional gradient interfaces, and in locations where sulfidic water is advected into oxygenated waters. Unlike its filamentous sister taxa (e.g., *Beggiatoa, Thioploca* etc.) *Thiomargarita* spp. is generally considered to be non-motile. As such, *Thiomargarita* ecotypes are thought to be adapted to habitats that experience temporal changes in geochemical conditions (Grünke et al., 2011). Indeed, some Namibian upwelling sediment ecotypes, such as *Ca*. T. nelsonii Thio36, experience anaerobic conditions for many months and infrequently experience access to oxygen and nitrate via methane eruptions. On the other hand, methane seeps such as Hydrate Ridge are much smaller habitats that are highly heterogenic both spatially and temporally and they are highly ephemeral. Perhaps the increased rate of genetic change, as well as phase and antigenic variation promoted by mobile genetic elements and metacaspases, increases *Thiomargarita's* adaptability to its environment such as developing a dimorphic lifestyle that includes attached and free-living stages. The attachment to mobile substrates such as marine animals (Bailey et al., 2011), may allow them to transit between oxygenated and sulfidic conditions. Would this lifestyle constitute a fastidious host-association typically seen in other bacteria with a high degree of genome instability? At this time we find it difficult to draw any definitive conclusions regarding this question. Too little is known about the ecology of *Thiomargarita* spp., their association with host organisms and how they themselves may be important hosts to other microorganisms. But the degree of plasticity in the Bud S10 genome does raise many questions about the evolutionary processes involved in keeping the genomes of most bacteria streamlined, while others organisms, such as eukaryotes and Bud S10, abound with mobile and/or repetitive genetic elements.

What is also particularly striking about the mobile genetic elements in Bud S10 genome, beyond the sheer number detected, is the presence of a group I intron in a protein-encoding gene that is thought to be essential to Bud S10's core metabolism. Arguably, group I introns have conserved motifs but typically not conserved sequences, and thus they can be difficult to detect. In eukaryotes, group I introns tend to occur in conserved regions in genes essential to the organism's core metabolism or DNA replication such as NADH dehydrogenase, DNA polymerase, recombinase A, and genes related to photosynthesis (Nielsen and Johansen, 2009; Swithers et al., 2009). Until recently, group I introns in bacteria were thought to be rare (Hausner et al., 2014). However, a recent study found that an estimated 15% or more the bacterial domain contains introns within their rRNA and that these strains likely escaped classic detection techniques in part due to biases in PCR (Brown et al., 2015). Similarly, the Family *Beggiatoaceae* has been difficult to detect via PCR based methods even when visibly present in a sample (Angert et al., 1998; Gillan et al., 1998; Edgcomb et al., 2002; López-García et al., 2003; Sekar et al., 2006; Stevens and Ulloa, 2008; Jones et al., 2015).

Previous work has shown that *Thiomargarita* spp. possess group IA3, ID, and IC1 introns in its 16S rRNA gene (Salman et al., 2012). We have expanded the number of ribosomal genes containing introns to the 23S gene and tRNAs but we have not determined the secondary structure or type of group I introns present in these genes. However, as we report here, a group IA2 intron homologous to introns in *Staphylococcus* bacteriophage genomes occurs in a gene essential to *Thiomargarita's* core metabolism of sulfur oxidation. Group I introns disrupting protein coding genes, particular those not associated with DNA synthesis (i.e., *recA* and *nrdE*), have very rarely been reported in bacteria (Hausner et al., 2014). A group IA2 intron has been found in thermophilic *Firmicutes* flagellin gene (Hayakawa and Ishizuka, 2009). The ribozymal activity was found to be temperature dependent, promoting motility under higher temperatures and thus the intron may function in a regulatory role for flagellin gene expression under lower temperatures (Hayakawa and Ishizuka, 2012). The only other reported examples group I introns are the IStrons found in a few Firmicutes and Fusobacteria (Tourasse et al., 2014). IStrons have been shown to have alternative splicing sites, which could lead to different protein variants (Hasselmayer et al., 2004). Some of these IStrons have been found without transposases (Tourasse et al., 2014). However, we did not find any reports of MITE-like sequences similar to IS elements within IStrons in these genomes. But some of these strains have been reported to possess MITEs (Kristoffersen et al., 2011) and/or to have the “decay products” of IStrons (Siguier et al., 2014). One question that remains to be resolved about the origin of the *dsrA* “MITEtron” is whether it was once an IStron that lost its transposase or did it result from a direct symbiosis between a MITE and an intron. Does the group I intron play a role is the dispersal of the MITE element? And how does the *dsrA* MITEtron affect the regulation and transcription of *dsrA*?

Typically in model sulfur bacteria, *dsrAB* is constitutively expressed, but the level of expression depends on the sulfur source, which indicates that its expression is tightly regulated (Eddie and Hanson, 2013; Weissgerber et al., 2013). Perhaps an endonuclease, not co-located with the intron, removes the entire MITEintron prior to transcription and thus, the MITEintron has no effect on the transcription of *dsrA*. Otherwise, the presence of the MITEintron could affect the transcription of *dsrA*. On the one hand, the long hairpin loop in the MITE region may function to slow down the RNA polymerase to facilitate time for folding and self-excision; but on the other hand, it could be detrimental to polymerase activity (Bikard et al., 2010). Furthermore, these scenarios do not take into account the possibility that the MITE sequence alone is recognized and acted upon by endonucleases, reverse transcriptases, transcriptional promoters etc., and how that could affect the expression of *dsrAB*.

The addition of long Pacific Biosciences reads to our metagenome assembly facilitated the reconstruction of one of the most complete genomes from representatives of the Family *Beggiatoaceae* to date. We suspect that mobile genetic elements in the genomes of sister taxa are primarily responsible for the high number of contigs typically seen in these assembles. Therefore, we suggest that long read sequencing should be employed in (meta-)genome sequencing in these strains. The production of a number of high quality genome assemblies would enable more detailed comparative analyses that would aid in the development and testing of hypotheses concerning their genetic potential and genome instability. Unfortunately, there are no cultivated strains of vacuolated *Beggiatoaceae*, but transcriptomics under *in situ* and in controlled incubation experiments could be employed to test and refine these hypotheses. We also believe that new approaches need to be employed to develop lab cultivars in order to improve our understanding of this clade. After all, members of the *Beggiatoaceae* include the largest bacteria in the world and this genome revealed they are even more enigmatic than previously thought.

## Author contributions

JB and BF conceived of this study, BF performed the DNA extraction and PCR screening of the single cell *Thiomargarita* samples. PF performed the Illumina DNA sequencing read assembly and analysis with the assistance of DJ, SJ, and GD in tetranucleotide binning, assembly and analysis. AK performed Pacific Biosciences DNA sequencing. BF performed the hybrid DNA sequencing read metagenome assembly, binning and all analyses reported herein except DJ and JB performed phylogenic analyses. MW and MM performed the assembly of the Thio36 genome. BEF wrote the manuscript (with input from all authors). All authors read and approved the final version of the manuscript.

## Funding

Portions of this research was supported by a Grant-in-Aid from the University of Minnesota Office of the Vice President for Research (#22025), and Alfred P. Sloan Foundation research fellowship, an Early Career Investigator in Marine Microbial Ecology and Evolution Award from the Simons Foundation and the U.S. National Science Foundation (NSF) grant EAR-1057119. The research cruise was funded by NSF grant OCE-0826254.

### Conflict of interest statement

The authors declare that the research was conducted in the absence of any commercial or financial relationships that could be construed as a potential conflict of interest.

## Supplementary material

Supplementary Material 1 **ESOM binning map. Contigs within black borders were manually screened post binning**.

Supplementary Material 2 **Enriched fasta file of *hupSL* and MITE sequence**.

Supplementary Material 3 **Amino acid alignment of arsenite oxidoreductase subunit A**.

Supplementary Material 4 **Spreadsheet of disrupted genes (.xls)**.

Supplementary Material 5 **Consensus sequences generated by RepeatModeler (.docx)**.

Supplementary Material 6 **Table of RepeatModeler generated consensus sequences (.docx)**.
