# Supplementary figures and images for: Single-Cell (Meta-)Genomics of a Dimorphic Candidatus Thiomargarita nelsonii Reveals Genomic Plasticity
Source: Front Microbiol. 2016 May 3;7:603. doi: 10.3389/fmicb.2016.00603 (PMC4853749; doi:10.3389/fmicb.2016.00603)

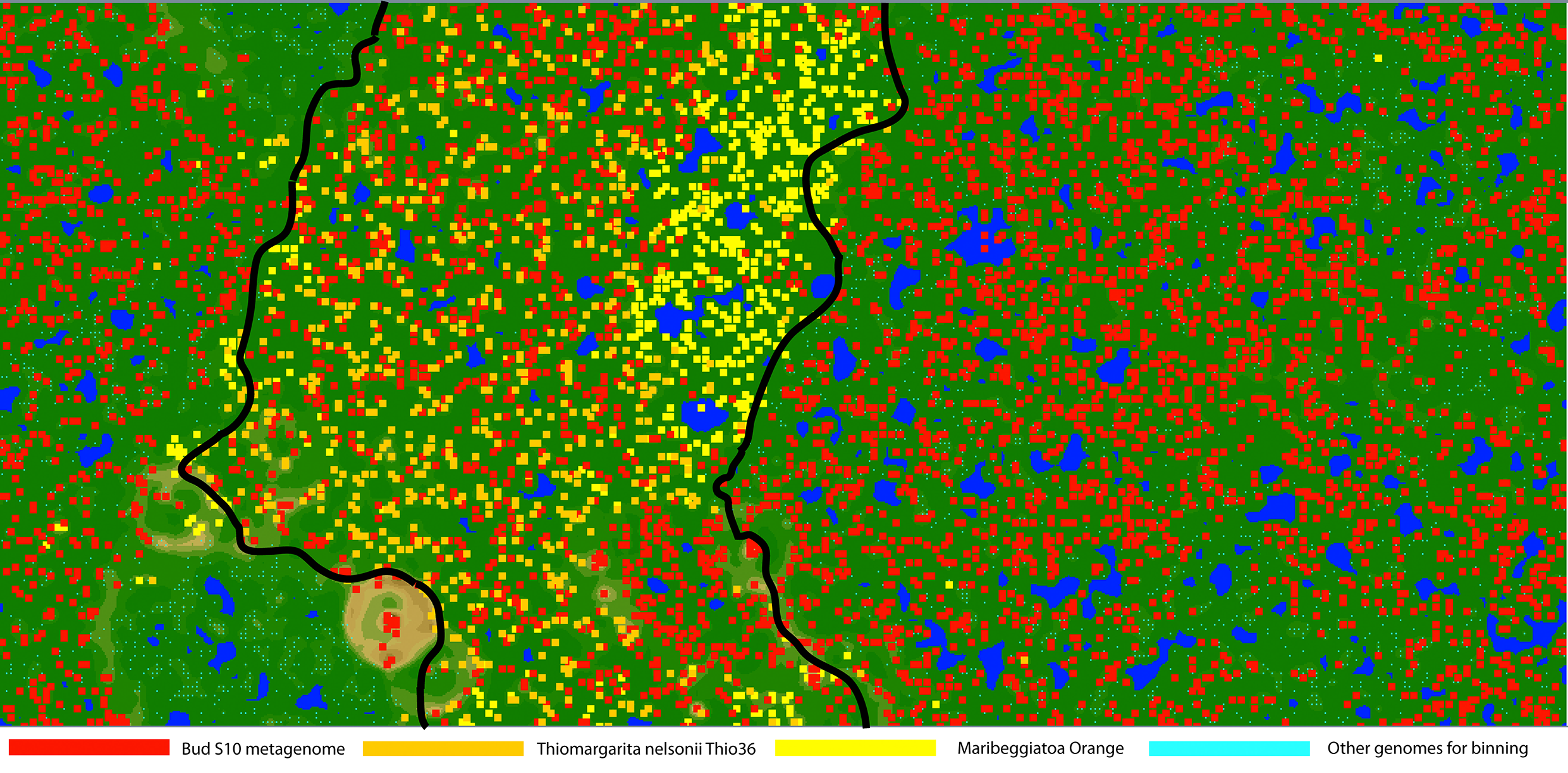

Supplement: Supplementary Material 1 — ESOM binning map. Contigs within black borders were manually screened post binning. [file Image2.TIF]
