# Supplementary material for: Single-Cell (Meta-)Genomics of a Dimorphic Candidatus Thiomargarita nelsonii Reveals Genomic Plasticity
Source: Front Microbiol. 2016 May 3;7:603. doi: 10.3389/fmicb.2016.00603 (PMC4853749; doi:10.3389/fmicb.2016.00603)
Supplement: Supplementary Material 3 — Amino acid alignment of arsenite oxidoreductase subunit A. [file Image1.PDF]

```
T-COFFEE, Version_11.00.8cbe486 (2014-08-12 22:05:29 - Revision 8cbe486 - Build 477)
Cedric Notredame
CPU TIME:0 sec.
SCORE=98
*
 BAD AVG GOOD
*
2601782023        :  98
2502841432        :  98
650365517         :  98
gi|12084499|pdb   :  89
gi|492768791|re   :  98
2551507680        :  98
2501726238        :  98
638944896         :  98
2547620395        :  98
2518887652        :  98
2600439513        :  98
2600436146        :  98
643498695         :  98
2601784055        :  97
2509285254        :  97
2511540676        :  98
647634471         :  98
2502307017        :  98
2510235734        :  98
cons              :   9

2601782023        MST----------------NYYLPED--SVPLPPKHAEVLTTCCDYCIVACAYKVYRWPLGS-
2502841432        MST----------------NYYLPED--RVPLPPKHADVLTTCCDYCIVACAYKVYRWPLGS-
650365517         MTT----------------NFYIPED--HAPLPPKNAEVITTACDYCIVACGYKVYRWPLEQ-
gi|12084499|pdb   ---------------------GCPND--RITLPPANAQRTNMTCHFCIVGCGYHVYKWPE-L-
gi|492768791|re   MAT----------------KYYLPED--HTPLPPKSADVMTTCCDYCIVACGYKIYRWPVGA-
2551507680        MTT----------------NFYIPED--HAPLPPKNAEKITTACDYCIVACGYNVYRWPLEQ-
2501726238        MTT----------------NFYIPED--HAPLPPTYAEVITTACDYCIVACGYKVYRWPIEK-
638944896         MTT----------------NFYIPED--HAPLPPKQAEVITTACDYCVVACGYKVYRWPLEM-
2547620395        MTT----------------NFYIPED--HAPLPPANAEIITTACDYCIVACGYKVYRWPLEK-
2518887652        MTT----------------NFYIPED--QAPLPPTDADVLTTACDYCVVACGFKVYRWPLEK-
2600439513        MAT----------------KYYLPED--HTPLPPKSADVMTTCCDYCIVACGYKIYRWPVGA-
2600436146        MAT----------------KYYLPED--HTPLPPKSADVMTTCCDYCIVACGYKIYRWPVGA-
643498695         MTQLSFNDKLALNDQSALNSQYLQPT--STLLPPASAEVITTACDYCIVACGYKVYRWPVSS-
2601784055        MN--------------------------KVPLPPPNAEIFTTACDYCIVACGYKVYRWPVGS-
2509285254        MAD----------------KTSPAEDSASVPLPPADAKVHTTVCDYCVVGCGYKVYTWPEG--
2511540676        MST----------------PYYIPGT--NIPLPPVDADVLSTACDYCIVACGYKIYRWPVKGG
647634471         MST----------------PYYIPGT--NIPLPPVDADVLSTACDYCIVACGYKIYRWPVKGG
2502307017        MST----------------PYYIPET--NIPLPPVDADVISTACDYCIVACGYKIYRWPVKGG
2510235734        MST----------------PYYVPEQ--SVPLPPPDADVISTACDYCIVACGYKVYRWPVAGG

cons                                             ***  *.  .  *.:*:*.*.:::* **


2601782023        GDGGPKASENAFGIDFPS--NPLQ-DWVAPAQHNVVM-HKSIPHNVVITPNKDATTVNTNGDS
2502841432        GDGGPKASENAYGIDFPS--NALQ-DWVAPAQHNVVM-HNMVPHNVVITPNKDAKTVNTNGDS
650365517         ANGGLKASDNAFGVDFPT--APLQ-AWVAPAQHNVVM-HNGKPHNIVIVPDKDTKAVNKTGDS
gi|12084499|pdb   EEGGRAPEQNALGLDFRKQLPPLA-VTLTPAMTNVVTEHDGARYDIMVVPDKAC-VVN-SGLS
gi|492768791|re   PDGGPKASENAFNTDFPS--GPLQ-AWVAPTQHNVVM-HKGRPHNVVIIPDKDSKVVNVGGDS
2551507680        ANGGMKASENAFGIDFPS--APLQ-AWVAPAQHNVIM-HDGKPHNIVVVPDKDSKVVNKTGDS
2501726238        ASGGQKASENAFGIDFPS--APLQ-AWVAPSQHNVVM-HLGKPHHIIIVPDKDSTVVNKMGDS
638944896         ANGGGKASENAFNVNFPS--GPLQ-AWVAPSQHNVVM-HKGKPHNIIVVPDKDAKVVNKMGDS
2547620395        ANGGQKASENAFGINFPS--APLQ-AWVAPSQHNVVM-HLGKPHHIVVVPDKDSTVVNKMGDS
2518887652        ANGGMKAEENAFGIAFPS--APLQ-SWVAPAQHNVVM-HNDKPHNIVIVPDKDTNAVNKNGDS
2600439513        PDGGPKASENAFNTDFPS--GPLQ-AWVAPTQHNVVM-HKGRPHNVVIIPDKDSKVVNVGGDS
2600436146        PDGGPKASENAFNTDFPS--GPLQ-AWVAPTQHNVVM-HKGRPHNVVIIPDKDSKVVNVGGDS
643498695         PSGGPAASENAFGVDFPV--HALQ-AWVSPQQHNIIS-HNGKPHHVVVVADKDIQVVNKGGDS
2601784055        REGGPKAHQNALGIDFPA--NIMSGQWVSPNKHNIVM-VNGKPHHILIMADADTEVVNVGGNH
2509285254        TSGGPAAKDNALGADFPV--QELSGQWLSPNAHSQCM-VDGRKHHVAIVADTDLQVVNKNGNH
2511540676        KVGGEKAAENAFGVDFPV--DPLG-PWVAPNQYNVVL-HKGEPHHVIIIPDKDTKHVNINGNS
```

```
647634471           KVGGEKAAENAFGVDFPV--DPLG-PWVAPNQYNVVL-HKGEPHHVIIIPDKDTKHVNINGNS
2502307017          KVGGENADENAFGVDFPV--DPLG-PWVAPNQYNVVL-HDGEPHHVIIIPDKDTEHVNLLGNS
2510235734          KVGGPAADQNAFGEDFPV--SPLG-AWVAPNQHNIVL-HNGEPHHVVIIPDKDTDFVNYTGNS

cons                  **  . :** .  *      :    ::*   .         :.: : .:     **  *


2601782023          SIRGGLIAQKCYNPKTPTRDRLSTPLMRIYGILQPVPWDFALDVAAEVAKHVIAKHGANAYSV
2502841432          SMRGGLIAQKCYNPKTPTRDRLTTPMLRIYGILQPVPWDFALDIAAEVAKHVIAKHGANAYSM
650365517           SMRGGLLAQKLYNPSTGTRDRLTQPLVRIGGSLQPVPWEFALDIAAQVGRHVIDNHGANAYGV
gi|12084499|pdb     STRGGKMASYMYTPTGDGKERLSAPRLYAADEWVDTTWDHAMALYAGLIKKTLDKDGPQGVFF
gi|492768791|re     SIRGGCIAQKCYNPDKPTNDRLTSPLVRINGTLQPVSWDFALDIAADVAKHVIKEHGANAYSV
2551507680          SMRGGLLAQKLYNPSTGTRDRLTQPLVRIGGSLQPVPWDFALDIAAEVGRHVIDTHGTNAYGV
2501726238          SMRGGLLAQKVYNPATGTRDRLTQPLVRIGGSLQPVPWDFALDIAAEVGSYVIDKHGTNAYCV
638944896           SMRGGLLAQKVYNPSTPTRDRLTQPLVRIGGSLQPVTWAFALDIAAAVGEHIINQHGANAYCV
2547620395          SMRGGLLAQKVYNPSTGTRDRLTQPLVRIGGSLQPVPWDFALDIAAEVGSYVIDKHGTNAYGV
2518887652          SMRGGLIAQKVYNPSTGTRDRLTSPLMRIGGTLQPVSWDMALNVAAAVGRHVLDKHGADAYGI
2600439513          SIRGGCIAQKCYNPDKPTNDRLTSPLVRINGTLQPVSWDFALDIAADVAKHVIKEHGANAYSV
2600436146          SIRGGCIAQKCYNPDKPTNDRLTSPLVRINGTLQPVSWDFALDIAADVAKHVIKEHGANAYSV
643498695           SVRGGLLARKLYNPETPTKDRLKQPLVRINGQLQPVEWDFALDIAAQVGRHVIDTHGSNSYCV
2601784055          SIRGGTIAQKCYNPKTLTKDRLKYPMLRVNGSLRRISWEMAFDIMAEVSQHVIDKYGKEAWAM
2509285254          SVRGGCLSKKVYSPDGPTSDRLKNPMMRIGGELKPVSWEMAIDVMAQVSQYIIDKHGVNAWGM
2511540676          SIRGGALAKKVYNPQTPTRDRLKSPMIRMFGTLMPVTWDLALEVAAEVGKHVIKKHGENAFCV
647634471           SIRGGALAKKVYNPQTPTRDRLKSPMIRMFGTLMPVTWDLALEVAAEVGKHVIKKHGENAFCV
2502307017          SIRGGALAQKVYNPQTPTRDRLKSPMIRMFGVLMPVTWDFAMEIAAEVGKHVIRTHGENAYCV
2510235734          SIRGGALAQKVYNPQTPTRDRLKSPMVRMFGVLMPVTWDFALDIAAEVGDHVLKKHGTNAYGV

cons                * ***  ::    *.*     :**. * :   .      *  *: : * :    :   * :.  .


2601782023          KTYSY----QFIENTYAITKYALRHINTASFTFHDTPSD-VTSTPGFRDAGFDNFGPSYDDWA
2502841432          KTYSY----QYIENTYAITKFALRHINTASFTFHDTPSD-VTSTPGFRDAGFDNFGPSYDDWG
650365517           KTFSY----QYIENTYAITKYALRHVNTANFTFHDTPSD-VTSTPGFRDAGFDNFGPSYDDWG
gi|12084499|pdb     SCFDHGGAGGFENTWGTGKLMFSAIQTPMVRIHNRPAY-NSECHATREMGIGELNNAYEDAQ
gi|492768791|re     KTYSY----QYFENTYAIKKFARRHIKTAAFTFHDTPSD-VTSTPGFRDAGFDNFGPAYKDWG
2551507680          KTYSY----QYIENTYAITKYALRHVNTANFSFHDTPSD-VTSTPGFRDAGFDNFGPSYDDWG
2501726238          KTYSY----QYIENTYAITKYALRHINTANFSFHDTPSD-VTSTPGFRDAGFDNFGPSYDDWG
638944896           KTYSY----QYIENTYAVTKYALRHLNTANFSFHDTPSD-VTSTPGFRDAGFDNFGPSYDDWR
2547620395          KTFSY----QYIENTYAITKYALRHINTANFSFHDTPSD-VTSTPGFRDAGFDNFGPSYDDWG
2518887652          KTYSY----QYIENTYAITKYAFRHVNTPNFAFHDTPSD-VTSTPGFRDAGFDNFGPSYDDWG
2600439513          KTYSY----QYFENTYAIKKFARRHIKTAAFTFHDTPSD-VTSTPGFRDAGFDNFGPAYKDWG
2600436146          KTYSY----QYFENTYAIKKFARRHIKTAAFTFHDTPSD-VTSTPGFRDAGFDNFGPAYKDWG
643498695           KTYSY----QYIENTYAITKYALRHVNTANFSFHDTPSD-VTATPGFRDAGFDNFAPSYDDWG
2601784055          KMFSY----QFWENTYALTKLALRSIRTAAFAVHDQPTGHGSDTPGLSDAGIDPFSAAYEDWK
2509285254          KTFSY----QYFENTYAISKLAFESIKTPAYAPHDKPGP-GADTAGIDDAGFNPFSASYDDWG
2511540676          KTFSY----GYMENTYAISKYALRSVSTANFTFHDTPSD-VTSTPGFRDAGFDNFGPSYQDWK
647634471           KTFSY----GYMENTYAISKYALRSVSTANFTFHDTPSD-VTSTPGFRDAGFDNFGPSYQDWK
2502307017          KTFSY----GYMENTYAITKYALNSVNTANFTFHDTPSD-VTSTPGFRDAGFDNFGPSYEDWR
2510235734          KTFSY----GYMENTYAITKYALRHINTANFTFHDTPSD-VSSTPGFRDAGFDNFGPSYDDWA

cons                . :.:       ***:.  *     : *.    *: *    :   .  : *:. :  :*.*


2601782023          SADVLMCLGTDPYETKTILFTQHIMPGVYNG----------------MKLIMVNPRETAGVAF
2502841432          NADVLMCCGTDPYETKTILFTQHIMPGIQHG----------------MKVIMLNPRETTGVAY
650365517           AADTLMICGTDPYETKTIIFTQFIMPAVRRG----------------MKTVILNPRETAGIAW
gi|12084499|pdb     LADVIWSIGNNPYESQTNYFLNHWLPNLQGATTSKKKERFPNENFPQARIIFVDPRETPSVAI
gi|492768791|re     DADVLMICGTDPYETKTMIFTQFIKPAIDRG----------------QKTIWLNVRETAGIAY
2551507680          AADTLMICGTDPYETKTIIFTQFIMPAVQRG----------------MKTVILNPRETAGIAW
2501726238          AADTLMVCGTDPYETKTILFTQFMMPAIQRG----------------MKTVILNPRETAGIAY
638944896           AADTLMICGTDPYETKTIIFTQYIMPAIQRG----------------MKTVILNPRETAGIAY
2547620395          AADTLMVCGTDPYETKTILFTQFIMPAIQRG----------------MKTVILNPRETAGIAY
2518887652          AADVLMIAGTDPYETKTILFTQYIMPAVRRG----------------MKTIILNPRETAGIAW
2600439513          DADVLMICGTDPYETKTMIFTQFIKPAIDRG----------------QKTIWLNVRETAGIAY
2600436146          DADVLMICGTDPYETKTMIFTQFIKPAIDRG----------------QKTIWLNVRETAGIAY
643498695           AADTLMICGTDPYETKTIIFTQFIMPAVHRG----------------MKTVILNPRETAGIAW
```

```
2601784055        SADVLFISGTDPFESKTIIFTEWIMKGILNG----------------MKVIFVVPRKTTGVAF
2509285254        KADVLFISGSDPYESKTVVFTDWMMKG---D----------------KKIIMVMPRKTTGAAW
2511540676        DAETLMVCGTDPYESKTILFTDWMMPAIQNG----------------QKTIFMIPRKTAGVAY
647634471         DAETLMVCGTDPYESKTILFTDWMMPAIQNG----------------QKTIFMIPRKTAGVAY
2502307017        DAETLLVCGTDPYETKTILFTDWMMPGIQNG----------------QKAIFMVPRKTGGIAY
2510235734        EADTLMMCGTDPYESKTILFTDYIMPAIQGG----------------QKTIFMLPRRTGGTAF

cons               *:.:   *.:*:*::*  * :                          : : :  *.* . *


2601782023        AKKMG----GLHLDVYPGTDTVLLGAIARIVLENGWQDNEWLKKWVNNKWESNSGFGQGTRNT
2502841432        AKKMG----GLHLDLYPGTDTVLLGAIARIVLENGWEDQEWLKKWVNNKWESDSGFGQGTRNT
650365517         LKQQG----GLHIDLNPGSDNLVVGAIIRVILENGWQDSEWIQNWVNNKWESSSGFGQGTRNT
gi|12084499|pdb   ARHVAGNDRVLHLAIEPGTDTALFNGLFTYVVEQGWIDKPFIEA-------------------
gi|492768791|re   AKSRGN---ALFLQVNPGTDTPVLGAISRIILENGWEDKDWIKHWVNDKWGSSSGFGQGTRNT
2551507680        LKKQG----GLHIDLNPGSDNLVVGAILRVIIENGWEDSEWINKWVNNKWETSSGFGQGTRNT
2501726238        MKKHG----GLHIDLNPGSDNLVIGAILRVIMDNGWQDDEWIDKWVNNKWESNSGFGQGTRNT
638944896         MKKHG----GLHLDVNPGSDTLVVGAIARIIMQNDWQDKEWISQWVNNKWESNSGSGQGTRNT
2547620395        MKKHG----GLHIDLNPGSDNLVVGAILRVIMDNGWQDDEWIDKWVNNKWESNSGFGQGTRNT
2518887652        LKTQG----ALILDVYPGTDTLVLGAIARVIMENGWEDSEWIQKWVNSKWESSSGFGQGTRNT
2600439513        AKSRGN---ALFLQVNPGTDTPVLGAISRIILENGWEDKDWIKHWVNDKWGSSSGFGQGTRNT
2600436146        AKSRGN---ALFLQVNPGTDTPVLGAISRIILENGWEDKDWIKHWVNDKWGSSSGFGQGTRNT
643498695         LKAQG----GLHLDINPGSDNLVIGAIARVILEQGWQDNEWIENWVNNKWESSSGFGQGTRNT
2601784055        GEKMG----GLWLDIIPGTDTILQMALARYIIENGWEDSEFLSKYINNRTERD----------
2509285254        AEKNG----GLFLPIIPGTDTVLHLALARLILENNWQDDEFIKDFVASRWEIDSGFGRGTRNT
2511540676        AEKNG----GLWLDIQPGTDLLVVNAIARVIIENGWQDAEWIRDWVNNKWESSSGFGQGTRNT
647634471         AEKNG----GLWLDIQPGTDLLVVNAIARVIIENGWQDAEWIRDWVNNKWESSSGFGQGTRNT
2502307017        AEKNG----GMWLDIQPGTDLLVVNAIARVIVENGWEDSDWIQKWVNNKWESSSGFGQGTRNT
2510235734        AEKNG----GIVLDIQPGTDLPVVLAIARVIVENGWQDDAWIEQWVNNKWESSSGFGQGTRNT

cons               .  .     : : :  **:*  :  .:   ::::.* *  ::


2601782023        PWQWRTTWGKFQTKGF---EDYKEWLLSQPEYELDKAAELTGVDAAKIRTAAEWIAKPKADGT
2502841432        PWQWRTTWGKFQTKGF---EDYKEWLLSQPEYELEKAAEITGVDPEKIKTAAEWIAKPKEDGS
650365517         PWQWRTTWGKFQTNGF---EGYKKWNLEQKEFDPKYAAQVAGIDEKKIYQAAEMLAKPV-DGV
gi|12084499|pdb   -----------HTKGF---DDAVKT----NRLSLDECSNITGVPVDMLKRAAEWSYKPKASGQ
gi|492768791|re   PWQWRTTWGKFQTNGVYGKKGYKAWVMSQKEYEPEVAAKIAGLDAKDLYKAAEWLTGA--GKA
2551507680        PWQWRTTWGKFQTDGF---EGYKKWNLEQKEYDPIYAAKMAGIDVDKIYKAAEMLAKPV-NGV
2501726238        PWQWRTTWGKFQTDGF---EDYKKWNQAQDEFDPKYAAKMAGIDVAKIYKAAEMMAKPV-NGV
638944896         PWQWRTTWGKFQTNGY---EDYKKWNLAQAEYDPDYAANMAGIEVKKLYQAAEMLAKPI-NGK
2547620395        PWQWRTTWGKFQTDGF---DDYKKWNQAQDEFDPKYAAKMAGIDVAKIYKAAEMMAKPV-NGV
2518887652        PWQWRTTWGLFQTDGF---EDYKTWNKEQDEYDPDYAARYAGIDVEKIYQAAEMLAKPK-DGK
2600439513        PWQWRTTWGKFQTNGVYGKKGYKAWVMSQKEYEPEVAAKIAGLDAKDLYKAAEWLTGA--GKA
2600436146        PWQWRTTWGKFQTNGVYGKKGYKAWVMSQKEYEPEVAAKIAGLDAKDLYKAAEWLTGA--GKA
643498695         PWQWRTTWGKFQTDGF---DDYKQWNLGQKEYAPEYAASIAGIDVEKIHKAAEMLAMPV-NGQ
2601784055        -------LEVFQANGF---SDYKGWILSYKYAELEIASEITGLSVDKIKQAAEMIAKPKPDGS
2509285254        PWQWRTTWGKLGA-SF---EQYKEWILSQELGELDRAAEITGIDADKIRQAAQMIAKPI-DGK
2511540676        PWQWRTTWGKFQTKGY---EDFVKWLMAQDEFEPTAAAEIAQIDVEKIYTAAEWMAKPREDGS
647634471         PWQWRTTWGKFQTKGY---EDFVKWLMAQDEFEPTAAAETAQIDVEKIYTAAEWMAKPREDGS
2502307017        PWQWRTTWGKFQTAGY---EDWKDWLLSQDEFAPDQAAEIAQIDVQKIYTAAEWMAKPKEDGS
2510235734        PWQWRTTWGKFQTDGF---EDWKEWLLAQDYAVPEKAAEIARIDAQKIYTAAEWMAKPKEDGT

cons                          : .    .                .:  : :    :  **:    .  .


2601782023        RVKASFGIEKGFYWSNNT-GNTNAISSLATICGAGGRPGQVVGRFGGHQRGGRRGGKLPRNKS
2502841432        RPKSSFGIEKGFYWSNNT-GNTNAISSLATICGAGGRPGQVVGRFGGHQRGGQRGGGLPRNKS
650365517         RPKTSIGIEKGFYWSNNT-GNTNAISSLATIVGAGGREGRVVGRFGGHQRGGQSGGKLPRNKS
gi|12084499|pdb   APRTMHAYEKGIIWGNDNYVIQSALLDLVIATHNVGRRGTGCVRMGGHQEGYTRP--------
gi|492768791|re   RPKASFGIEKGFYWSNNT-GNTNAISSIATICGAGGRPGQVVGRFGGHQRGGAGGGSYPRNRS
2551507680        RPKTSIGIEKGFYWSNNT-GNTNAISTLATVVGAGGREGQVVGRFGGHQRGGQSGGKLPRNKS
2501726238        RPKTSIGIEKGFYWSNNT-ANTNAISTLATVVGAGGREGRVVGRFGGHQRGGQSGGKLPRNKS
638944896         RPKTSIGIEKGFYWSNNT-GNTNAISTLATLVGAGGREGQVIGRFGGHQRGGQSGGKLPRNKS
2547620395        RPKTSIGIEKGFYWSNNT-ANTNAISTLATVVGAGGREGQVVGRFGGHQRGGQSGGKLPRNKS
2518887652        RPKASIAIEKGFYWSNNT-ANTNAVASLATIIGTGGREGQVIGRLGGHQRGGVSGGKLPRNKS
```

```
2600439513          RPKASFGIEKGFYWSNNT-GNTNAISSIATICGAGGRPGQVVGRFGGHQRGGAGGGSYPRNRS
2600436146          RPKASFGIEKGFYWSNNT-GNTNAISSIATICGAGGRPGQVVGRFGGHQRGGAGGGSYPRNRS
643498695           RVKASIGIEKGFYWSNNT-GNTNAIASLATIIGTGGREGRVIGRFGGHQRGGLAGGKLPRNKS
2601784055          RKKTSFGIEKGNYWSNNY-LNTASFASLGLICGAGNRPGQVISRFGGHQRGMMPGGKYPIEDA
2509285254          HPKTSFALEKGNYWSNNY-LNTASYAALATICGAGNREGRVVSRLGGHQRGWMGAASYPRVYS
2511540676          RPKTSIMIEKGFYWSNNT-GNTNAISSLGIICGCGGRPGQVIGRAGGHQRGGLKGGSYPRNKS
647634471           RPKTSIMIEKGFYWSNNT-GNTNAISSLGIICGCGGRPGQVIGRAGGHQRGGLKGGSYPRNKS
2502307017          RPKTSVMIEKGFYWSNNT-GNTQAISALGIIVGAGGRPGQVIGRAGGHQRGGLRGGKYPRNKS
2510235734          RPKTSIMIEKGFYWSNNT-GNTQAISALGIAVGAGGRPGQVIGRAGGHQRGGLRGGGYPRNKS

cons                  ::    ***  *.*:      :   :        .* *     * ****.*


2601782023          PEKVPGRRRRALDTDRWLVSGHTRMAHVIGTTWIQAMSGSQGLQQRFHE---LVVTNPHQVRT
2502841432          PEKVPGRRRRALDTDRWLVSGHTRMAHVVGTTWIQAMCGSQGLQKAFQE---LVVNNPHQVRT
650365517           PEKVPGRRRRSIDTDRYLYSGHTRFAHVIGTTWIQAMCGSQGLQKKFWE---LTTANPHQVYS
gi|12084499|pdb     --PYPGDKK--IYIDQELIKGKGRIMTWWGCNNFQTSNNAQALREAILQRSAIVKQAMQKARG
gi|492768791|re     PEKVPGRRRRSLDTDRWLYSGHTRMAHVIGTTWIQAMTGSNGLHAKFEE---LVTNNPNQVTS
2551507680          PEKVPGRRRRAIDTDRYLYSGHTRFAHVIGTTWIQAMCGSQGLQKKFWE---LTTANPHQVYS
2501726238          PEKLPGRRRRALDTDRYLYSGHTRFAHVIGTTWIQSMCGSQGLQKKFWE---LVTSNPHQVYS
638944896           PEKVPGRRRRALDTDRYLFSGHTRFAHVIGTTWIQSMCGSQGLQKKFWE---LTIANPHQVFS
2547620395          PEKVPGRRRRALDTDRYLYSGHTRFAHVIGTTWIQSMCGSQGLQKKFWE---LVTSNPHQVYS
2518887652          PEKLPGRRRRALDTDRWTYSGHTRFAHVIGTTWIQSMAGSQGLQRKFWE---LVSANPNQVHS
2600439513          PEKVPGRRRRSLDTDRWLYSGHTRMAHVIGTTWIQAMTGSNGLHAKFEE---LVTNNPNQVTS
2600436146          PEKVPGRRRRSLDTDRWLYSGHTRMAHVIGTTWIQAMTGSNGLHAKFEE---LVTNNPNQVTS
643498695           PEKVAGRRRRSIDTDRYLYSGHTRMAHVIGTTWIQAMCGSYGLKEKFTD---LTTRNPHQAMR
2601784055          VEKFPGRKIKAIDLDRWVEAGKVRFAWVVGTTWLQAMAGSQGLRKSFEK---MTRDNPYQITR
2509285254          PEKGPGRRKKEMDLDRWVEAGKLRFAWVIGTTWCQAMAASKELMNRFKA---MTTDSEHQIAQ
2511540676          PEKLPGRRRRAMDTDRYLMSGHTRFAHVIGNTWIQSMCGSQSLAAKFEE---LTVQNPNQVRS
647634471           PEKLPGRRRRAMDTDRYLMSGHTRFAHVIGNTWIQSMCGSQSLAAKFEE---LTVQNPNQVRS
2502307017          PEKLPGRRRRAMDTDRYLMSGHTRFAHVIGNTWIQSMCGSQSLAAKFNE---LTVQNPHQIRS
2510235734          PEKLPGRRRRAMDTDRYLMSGHTRLAHVIGTTWVQAMCGTQSLQTKFDE---LTTRNPHQVNS

cons                     .* :   :  *:     *: *:     *  .  *:    :  *    :      :.      :


2601782023          YDKQEIIDTLKARADSGGMVVINQDIYLRNPIGAQFSDIVFPAATWGEEDFMRANGERRLRLY
2502841432          YDKQEIIDTLKARADSGGMVVIDQDIYLRNPIGGQFADIIFPAATWGEEDFMRANGERRLRLY
650365517           YDKQEIIDTLKKRADSGGMVVINQDIYLRDPIGAKFADIVFPAATWGEVDFMRANGERRLRLY
gi|12084499|pdb     ATTEEMVDVIYEATQNGGLFVTSINLYPTK--LAEAAHLMLPAAHPGEMNLTSMNGERRIRLS
gi|492768791|re     WEKDEIIKTLKARADSGGTVVIDQDIYLRDPIGARFADIVFPAATWGEEDLARANGERRIRLY
2551507680          YDKQEIIDTLKKRADSGGMVVINQDIYLRDPIGAKFADIVFPAATWGEVDFMRANGERRLRLY
2501726238          YDKQEIIDTLKKRADSGGMVVINQDIYLRDPIGAKFADIVFPAATWGEVDFMRANGERRLRLY
638944896           YDKQEIIDTLKARADSGGMVVVNQDIYLRDPIGAKFADIVFPAATWGEEDFMRANGERRLRLY
2547620395          YDKQEIIDTLKKRADSGGMVVINQDIYLRDPIGAKFADIVFPAATWGEVDFMRANGERRLRLY
2518887652          YDEKGIIETLKARCDSGGMVVINQDIYLRDPIGAKFADIVFPAAGWGEENFIRANGERRLRLY
2600439513          WEKDEIIKTLKARADSGGTVVIDQDIYLRDPIGARFADIVFPAATWGEEDLARANGERRIRLY
2600436146          WEKDEIIKTLKARADSGGTVVIDQDIYLRDPIGARFADIVFPAATWGEEDLARANGERRIRLY
643498695           RDKQHIIDTLKKRADSGGMVVINQDIYLRDPIGKQFADIIFPAATWGEEDFMRANGERRLRLY
2601784055          LDKRHAIDMLKKRVDSQGMLVIHQDIYPVAPIGTEIADIVLPAAGWGEENFSRANGERRIRLY
2509285254          ADVAHAVDVLKKRVDDGGMFVVHQDIYMRGPMGSDFADIVLPAATWGEEDFTRCNGERRLRLY
2511540676          YDKQTIIDTLKKRVDSGGMVVVNQDIYLVDPIGARFADIVFPAAGWGEDTFTRANGERRLRLY
647634471           YDKQTIIDTLKKRVDSGGMVVVNQDIYLVDPIGARFADIVFPAAGWGEDTFTRANGERRLRLY
2502307017          FDKQEIIDSLKRRVDSGGMVVVNQDIYLVDPIGARYADIVFPAAGWGEDNFTRANGERRIRLY
2510235734          FEKQDIIDTLKRRADSGGMVVWNQDIYLVDPIGARYADIVFPASGWGEETFTRANGERRLRLY

cons                     :. :     :. * .*   ::*           :.:::**:  **  :    *****:**


2601782023          QKFYDAPGDAKPDWWIIAQLAKRM--------------GFDGFDWKNSNDVAEESSRFSRGGR
2502841432          QKFYDAPGDAKPDWWIIAQLAKRM--------------GFDGFDWNNSNDVAEESSRFSRGGR
650365517           QKFADAPGSAQPDWWIISQLATRM--------------GYDGFDWNNSNDVAEEASRFSRGSR
gi|12084499|pdb     EKFMDPPGTAMADCLIAARIANALRDMYQKDGKAEMAAQFEGFDWKTEEDAFNDG--FRRAGQ
gi|492768791|re     QKFYDAPGDAKPDWWIIAGLAKRM--------------GFEGFDWKDSNQVCEESSRSSRGNR
2551507680          QKFADAPGQAQPDWWIISQLANRM--------------GYDGFDWQNSNDVAEEASRFSRGSR
2501726238          QKFNDAPGQAQPDWWIVGQLAKRM--------------GFDGFDWENSNDVAEEASRFSRGSR
```

```
638944896          QKFNDAPGNAKPDWWIIAQLSKRM--------------GFDGFDWQNSNEVAEEASRFSRGSR
2547620395         QKFNDAPGQAKPDWWIVGQLAKRM--------------GFDGFDWENSNDVAEEASRFSRGSR
2518887652         QKFNDAPGEARPDWWIISQLAQRM--------------GFDGFDWENSNEVAEEAARFSRGSR
2600439513         QKFYDAPGDAKPDWWIIAGLAKRM--------------GFEGFDWKDSNQVCEESSRSSRGNR
2600436146         QKFYDAPGDAKPDWWIIAGLAKRM--------------GFEGFDWKDSNQVCEESSRSSRGNR
643498695          QKFADAPGQAKPDWWIVAQMASRM--------------GYDGFDWQNSNDVAEESSRFSRGSR
2601784055         SKFNDAPGEAKPDWWIAAKFAQRM--------------GFSGFDWKDSNEIFEEAAWFGKSRR
2509285254         SKFCDAPGEAKPDWWIIAQFAQKM--------------GFKDYDWKDSNDVFEQAARFGRKGV
2511540676         PKFYDAPGEAKPDWWIVANLAKKM--------------GFKGYDWKDSNEVLEEAARFSRGSR
647634471          PKFYDAPGEAKPDWWIVANLAKKM--------------GFKGYDWKDSNEVLEEAARFSRGSR
2502307017         PKFYDAPGEARPDWWIIAQLATAM--------------GYKGFDWKNSNEVLEEGARFSRGSR
2510235734         PKFYDAPGEAKPDWWIIANLAKKM--------------GFEGFDWKNSNDVLEEGARHSRGSR

cons                ** *.** * .* * . :: :                :..:**: .:: ::.    :


2601782023         KAYHMLKIYAHRQGR-TLHEQLRLLGADGIQGPTFIN-E-------------------KGELK
2502841432         KAYHMIKVYAHRHGR-TLHEQLRLLGADGIQGPTFIN-D-------------------QGELT
650365517          KDFNMIKVAAHAEGK-TLHEKLREYGTDGIQGPVFYNYD-------------------TKQLV
gi|12084499|pdb    PGAPAIDSQGGSTGHLVTYDRLRKSGNNGVQLPVVSWDE-------------------SKGLV
gi|492768791|re    KAYHMIKVAAHAEGK-TLHQKLAELGTEGIQGPTFYNYE-------------------TGELL
2551507680         KDFNMIKVAAHAEGK-TLHEKLREYGTEGIQGPVFYNYD-------------------TKKLV
2501726238         KDFHMIKVAAHIEGK-TLHQKLREYGTEGIQGPVFYNYD-------------------TKKLV
638944896          KDFNMIKVAAHAEGK-TLHQKLKEYGTEGIQGPVFYNYD-------------------TKQLV
2547620395         KDFNMIKVAAHAEGK-TLHQKLKEYGTTGIQGPVFYNYD-------------------TKELV
2518887652         KDFFMVKVAAHKEGK-TLHEKMREFGTDGIQGPVFYNYD-------------------TGKLV
2600439513         KAYHMIKVAAHAEGK-TLHQKLAELGTEGIQGPTFYNYE-------------------TGELL
2600436146         KAYHMIKVAAHAEGK-TLHQKLAELGTEGIQGPTFYNYE-------------------TGELL
643498695          KDFNMIKVAAHAEGK-TLHQKLAEFGTEGIQGPVFYNYD-------------------TKKLI
2601784055         TSYLPLVWYAKDQGK-RGHDLLREYGTTGIQAPVRY-E--------------------DGQLI
2509285254         LNYHPLVYYAKKSGK-RGHELLREMGTHGIQTPVRYRETLTESDEYKNYAGYYDDPNVAGAIV
2511540676         KDFFMLKVAAQKEGK-TLHEKFAEFGTDGIQGPVVML-E-------------------DGTLQ
647634471          KDFFMLKVAAQKEGK-TLHEKFAEFGTDGIQGPVVML-E-------------------DGTLQ
2502307017         KDFFNVKIVAQREGK-TLHEKLAEFGTNGIQGPVLLQ-D-------------------DGTLV
2510235734         KDFNMVHVAAKREGK-TLHEKFGEFGTNGIQGPVLML-A-------------------DGSLE

cons                    :   .   *:   :: :   *  *:* *.                            :


2601782023         GTKRLHDTTMTAE--MIAERYGDNGPGEANMVNKKMTHFNTQTGKVNLQKHPWSLFSDFWTWL
2502841432         GTKRLHDIFMTPE--MSAERYGADGPGGANMVNKKMSHFNTQTGKVNLQKHPWSLFSDYWAWL
650365517          GTKRLHDTELSEQ--ALAEKGLTDGPQGGNVLKKQLTHFNSQTGKVNLQKHPWDLFSDFYAWL
gi|12084499|pdb    GTEMLYTE----------------------------GKFDTDDGKAHFKPAPWNGLPATVQQQ
gi|492768791|re    GSVRLHNTTMTQE--QIEKEGR---TKGANMINKKGTHFNSQTGRVNIQKHPWSLYADYWEWM
2551507680         GTKRLHDTEMTLD--TLAEKGLANGPQGANVLKKQLTGFNSQTGKVNLQKHPWDLFSDFYAWL
2501726238         GTKRLHDTEMTAE--TLAEKGLANGPQGQNVLKKQLTAFNSQTGKVNLQKHPWDLFSDFYAWL
638944896          GTKRLHDTQMSVE--TLAEKGLTDGPQGANVLKKQLTGFNSQTGKINMQKHPWGLFSDFYAWI
2547620395         GTKRLHDTEMTAE--TLAEKGLTNGPQGQNVLKKQLTAFNSQTGKINLQKHPWDLFSDFYAWL
2518887652         GTKRLHDVALATDAKRLEVMGLTNGAVGANVLGKKMTGFNTQTGKINLQKHPWGLFSDLHKWL
2600439513         GSVRLHNTTMTQE--QIEKEGR---TKGANMINKKGTHFNSQTGRVNIQKHPWSLYADYWEWM
2600436146         GSVRLHNTTMTQE--QIEKEGR---TKGANMINKKGTHFNSQTGRVNIQKHPWSLYADYWEWM
643498695          GTKRLHDTEMSWD--DLVDKGLQDGPQGANIMRKQLTQFNSQTGKVNLQKHPWDLFSDFHRWL
2601784055         GTTRLHDSTLKLG--------T-SYNLTAN-NAKWLTEFKTKTGRANLLKTPWELFSDFYEFI
2509285254         GTKRLHDPDIDMG--------V---PEGPTVHMKWMSAFGSHSGKAVLHKTPWDLFGDFYDRI
2511540676         GTKRLHDTTRELS---------ATGPSGSNRYNKKLTHFNSQTGKCNIQKSPWSLFSDYWAWM
647634471          GTKRLHDTTRELS---------ATGPSGSNRYNKKLTHFNSQTGKCNIQKSPWSLFSDYWAWM
2502307017         GTKRLHDTERKLP---------ADGPSGANRLGKKLTHFNSQTGKCNIQKSPWSLFSDYWAWL
2510235734         GTKRLHDVNRVLP---------DTGPQGANVFNKKLTHFNSQTGKCNIQKAPWDLFSSYWEWM

cons               *:  *:                                *  :.  *:  :   **


2601782023         KPKDDELWFSNGRINEIWQSGFDDVERRPYITQRWPENFVEVHPDDAKARGIESGDYVMMYSD
2502841432         KPQGEELWMTNGRINEVWQSGFDDQQRRPYITQRWPENFVEIHPDDAKARGIESGDYVMLHSD
650365517          QPKGEELWFSNGRINEIWQSGFDDVERRAYVIQRWPENWVEVHPDDAKQRGIESGDQVMMYSD
gi|12084499|pdb    KDK-YRFWLNNGRNNEVWQTAYHD-QYNSLMQERYPMAYIEMNPDDCKQLDVTGGDIVEVYND
```

```
gi|492768791|re    SPKEDELWHTNGRINEVWQSGFDDIERRAYITQRWPENFTEIHPDDAAARGIESGDRVMLYSN
2551507680         QPKEDELWFSNGRINEIWQSGFDDVERRAYVIQRWPENWVEINPEDAKARGIESGDQVMMYSD
2501726238         QPREDELWFSNGRVNEIWQSGFDDVERRAYVQQRWPENWVEIHPEDAKKRGIESGDQVMMYSD
638944896          KPKGDELWFSNGRVNEIWQSGFDDVERRAYTAQRWPENWVEIHPDDAKKRGIESGDQVMMYSD
2547620395         QPREDELWFSNGRVNEIWQSGFDDVERRAYTAQRWPENWVEIHPEDAKRRGIESGDQVMMYSD
2518887652         QPKNDELWFSNGRINEIWQSGFDDVERRAYTAQRWPENWVEIHPDDAAERGIEAGDRVMMYSD
2600439513         SPKEDELWHTNGRINEVWQSGFDDIERRAYITQRWPENFTEIHPDDAAARGIESGDRVMLYSN
2600436146         SPKEDELWHTNGRINEVWQSGFDDIERRAYITQRWPENFTEIHPDDAAARGIESGDRVMLYSN
643498695          TPKQDELWFSNGRINEIWQSGFDDTERRPYITHRWPENWIEVHPEDADKRGIEAGDEILVYSE
2601784055         KPKADELWVTSGRINEFWQSGFDDQQRRPYLKQRWPDNFLEIHPDDAKVRGIESGDMLLIESD
2509285254         KPKGDELWVTCGRINEIWQTMFDD-SRREYIKQRWPEQCIEIHPNDAKRFGIESGDEVLVTND
2511540676         KPKDDELWVTSGRINERWQSGYDD-RRRPYIVQRWPENWVEIHPDDAKERGIENGDYVMLYSD
647634471          KPKDDELWVTSGRINERWQSGYDD-RRRPYIVQRWPENWVEIHPDDAKERGIENGDYVMLYSD
2502307017         KPKDDELWVTSGRINERWQSGYDD-RRRPYIVQRWPENWVEIHPDDAAERGIENGDYVMMYSD
2510235734         KPREDEIWISSGRINERWQSGYDD-RRRPYIVQRWPENWIELHPSVAEARGIESGDYVQVYSD

cons                 :  .:* . ** ** **: :.*   .    .*:*    *::*. .   .:  ** : : .:


2601782023         RVAAQ----------------------------------------------------------
2502841432         RVAAH----------------------------------------------------------
650365517          RVANF----------------------------------------------------------
gi|12084499|pdb    FGST-----------------------------------------------------------
gi|492768791|re    RVPVH----------------------------------------------------------
2551507680         RVANF----------------------------------------------------------
2501726238         RVANF----------------------------------------------------------
638944896          RVPSF----------------------------------------------------------
2547620395         RVANF----------------------------------------------------------
2518887652         RVPVF----------------------------------------------------------
2600439513         RVPVH----------------------------------------------------------
2600436146         RVPVH----------------------------------------------------------
643498695          RVANF----------------------------------------------------------
2601784055         EVPIQVGGFSHKDAAVRGVVDDTYSKEAETPDLIMQQSVTPQPDRGFDEMMSADVESDLLLDS
2509285254         DVLIQ----------------------------------------------------------
2511540676         RIPVQ----------------------------------------------------------
647634471          RIPVQ----------------------------------------------------------
2502307017         RIPVQ----------------------------------------------------------
2510235734         RIPVQ----------------------------------------------------------

cons


2601782023         ----------------------KDTIIGVHNDH---FQFSELMKRGHIELSKAAVTAVAVVTP
2502841432         ----------------------KDTILGVHNDH---FQFASLMERGHIELSKAAVTAVAVLAP
650365517          ----------------------KDTILGVHGDD---FQFSKLMENGHIKLDKAAVTAVAIVTP
gi|12084499|pdb    -------------------------------------------------------FAMVYPVA
gi|492768791|re    ----------------------KNTIKGVHGKD---FQFSELMKNGHIALEKGATTAIAIVTP
2551507680         ----------------------KDTILGVHGDD---FQFSKLMENGHIKLDKAAVTAVAIVTP
2501726238         ----------------------KDTILGVKGDD---FQFSSLLKNGHIQLDKAAVTAVAIVTS
638944896          ----------------------KDTILGVEGDD---FQFSQLLDAGHIQLDKAAVTAVAIVTN
2547620395         ----------------------KDTILGVKGDD---FQFNSLLKNGHIQLDKAAVTAVAIVTS
2518887652         ----------------------KDTILGIDESH---FQFSTLMERGHIKLDTAAVTAVAIVTP
2600439513         ----------------------KNTIKGVHGKD---FQFSELMKNGHIALEKGATTAIAIVTP
2600436146         ----------------------KNTIKGVHGKD---FQFSELMKNGHIALEKGATTAIAIVTP
643498695          ----------------------KDTILGVESDD---FQFSNLMKNGHIQLDYAEIKAVAIVTS
2601784055         LSTRRVERRESRDHFDFSDGLGEDSALGIPWSDIKAMTFTELKKNGYIKLTKGSFKAVAIVTD
2509285254         ----------------------TSGFVAVHSND---ASYTSLEKNGHIRVGKGEMKAVAIVTD
2511540676         ----------------------TDTTVGIEGDD---FTFTKLMDEGHIELTEAAITAVAIVTP
647634471          ----------------------TDTTVGIEGDD---FTFTKLMDEGHIELTEAAITAVAIVTP
2502307017         ----------------------KDTILGVEGDD---FQFTKLMEHGHIELTEAAITAVAIVTP
2510235734         ----------------------TDTIVGVEGSD---FDFAELLKNGHIELTKGSITAVAIVTP

cons                                                                       *:.  .


2601782023         HIKKGMMYANMLDTRQPSNALSARVVDHISGNYNYKMGVAKVQKIGE-SQYKKEFRSMSFAPR
```

```
2502841432        HIKKGVLYTMFIDMRQPSNAIMPRVVDNISGNYNYKLGVVKINKLGE-SKYKNEFRSMSFAPR
650365517         AVKKGTLYANMIDMRQPSNALTTRVVDQISGNYNYKMGVANIKKIGE-SKYKNEFRSFSFAPR
gi|12084499|pdb   EIKRGQTFMLFGYVNGIQGDVTTDWTDRDIIP-YYKGTWGDIRKVGSMSEFK---RTVSFKSR
gi|492768791|re   HVKKGTLYSYFITQGQPSNALQGRVVDNISGNYNYKLGVCKIKNLGP-SEYKDEFRSMSFAPR
2551507680        AVKKGALYANMIDMRQPSNSLTTRVVDQISGNYNYKMGVAKIKKLGE-SKYKSEFRSFSFAPR
2501726238        ATKQGALYANMIDMRQPSNSLTTRVVDQISGNYNFKMGVSKIRKIGE-SKYKHEFRAFSFAPR
638944896         STKKGVLFANMIDMKQPSNSLTARIVDQISGNYNYKMGVANIKKIGA-SVYKEEFRSFSFAPR
2547620395        ATKKGALYANMIDMRQPSNSLTTRVVDQISGNYNFKMGVSKIRKIGE-SKYKHEFRAFSFAPR
2518887652        AVKKGVLYANMLDMRQPSNSLTVRVVDNISGNYNFKMGVAKVRKIGE-STYKKEFRSFSFAPR
2600439513        HVKKGTLYSYFITQGQPSNALQGRVVDNISGNYNYKLGVCKIKNLGP-SEYKDEFRSMSFAPR
2600436146        HVKKGTLYSYFITQGQPSNALQGRVVDNISGNYNYKLGVCKIKNLGP-SEYKDEFRSMSFAPR
643498695         ATKKGVLYCNMMDMKNPANALTTRVVDQISGNYNYKMGVAKIKKIGE-SKYKREFEGFSFAPR
2601784055        AIRPKVAFTYFLIPSSPANAVAPRVLDPVSQRYRYKLGKGRVRKIGE-SPYKKDLTQMSFKPR
2509285254        AVRPGVMWTNALMPGSPANSLVHRVPDPITNRYRFKLGKGKIKKTGE-SPHKSEFERLTFAPR
2511540676        ALKKGMLYMDFLHTAQPANALSGRVVDWISGNYNYKMGVGRIKKIGT-SPYKDSFRSMSFARR
647634471         ALKKGMLYMDFLHTAQPANALSGRVVDWISGNYNYKMGVGRIKKIGT-SPYKDSFRSMSFARR
2502307017        AVKKGLLYMDFLHTAQPANALSGRVVDWISGNYNYKMGVGRIRKIGT-SPYKTSYRSMSFARR
2510235734        AVKENLGYMDFLHTAQPANALSGRVVDWISGNYNYKMGVGKVRKMGV-SPYKNQFRSMSFARR

cons                :    :          . :     *       :*     :.: *  * .*     .:*  *


2601782023        NIT-
2502841432        NIT-
650365517         NIV-
gi|12084499|pdb   RF-G
gi|492768791|re   NIV-
2551507680        NIV-
2501726238        NIV-
638944896         NIV-
2547620395        NIV-
2518887652        NIV-
2600439513        NIV-
2600436146        NIV-
643498695         NIV-
2601784055        SII-
2509285254        TVVP
2511540676        DIA-
647634471         DIA-
2502307017        DIA-
2510235734        DIA-

cons               .
```
