# Supplementary material for: Single-Cell (Meta-)Genomics of a Dimorphic Candidatus Thiomargarita nelsonii Reveals Genomic Plasticity
Source: Front Microbiol. 2016 May 3;7:603. doi: 10.3389/fmicb.2016.00603 (PMC4853749; doi:10.3389/fmicb.2016.00603)
Supplement: Supplementary Material 5 — Consensus sequences generated by RepeatModeler (.docx). [file DataSheet1.DOCX]

*hupSL* with MITE sequence with IMG annotation. Green = *hupS,* Grey = frameshift of *hupL*, Black = unsure if amino acid coding, Blue = *hupL,* Red = MITE, ***Direct Repeat,* Inverted repeat**, and inverted repeat.

Based on amino acid alignments, both *hupS* and *hupL* appear to be incomplete genes with hupS abruptly terminating at the putative frameshift of *hupL* and vice versa.

>2601783460 NADH ubiquinone oxidoreductase, 20 Kd subunit [Thiomargarita nelsonii bud S10 Ga0063879 : Ga0063879_1132] (-)strand 1850..2638 (-)

ATGCCAACTTTATTATGGATACAAACCGGTGCTTGTAGCGGAGACACTATGTCTCTGCTCAACGCCGAAAACCCAGATTTGTTAGAAACCCTAGAAAATTATGCCATTGAACTCTTATGGCATCCTTCACTCTCGCTTGAATCGCCGCTAGAATTGAATACCATTATCAATCAAATCCTATCCGATGAAAAACAATTAACGATTCTGTGCATTGAAGGTGCAATACTAATGGGGCCAAAAGGGACTGGTATGTATGATACTTTTTTGGGCAGTGCAAAAAAAGACATTGTAGCCTCTTTATGTGATAAAGCCACTTTTGTAGCTGCAATGGGGACTTGTGCTGCCTTTGGCGGAATTCCCGCCGCTCCACCGAATCCAGCAACAGCAGTGGGCTTACAATGGTCCGGGGAAAGGCCCAAAGGGCTACTGAAACCCGATTGGCATTCGAGCGGTGGCTTACCCGTTATCAATATTGCCGGTTGCCCCGCCCATCCTAATACAATGGTTCAAACACTTATTGATCTCACCCTGCTGGGTAAATCACTTGAATTAGACGATCTCAATCGCCCCAAAGACTTTTACAATACGATGGTACACCAAGGTTGCACCAGAAACGAATACCATGAATACGATGTAGAAGAAACTTCTTTTGGTTTACCGTCGATTTTTGTAACCAGCGCTATGCGGAACATCATGACTATTCTCGTATCCTCAAAGCCTTTGAACCATTTAAAGGTCAATTTTACTGTGAAACAGTTCAATATACCAAGAAAATCCTATAAATTGTAG

>intergenic 1849…1815

CTATATTCGGTGGACAATGGCCACACTCTTCCTAT

>2601783459 hypothetical protein [Thiomargarita nelsonii bud S10 Ga0063879 : Ga0063879_1132] (-)strand 1539..1814 (-)

ATGATTCCCGGCGGCGTCACCTCGGTGCCGAGTCAAAGGGATATAATTCGTGCCCTTTCTATTTTGGACACTCAGATTAAATGGTATGAAAATTCTATTCTAGGCTGCTCAATCGAAGAGTGGCTCTCCCTAAAAAGTGTAGAAGATTTTAACCATTGGCTTGAAAAGAAACGAGCTACTCAAGACACAACGGCGACTATGTGGTCAAACCTTCTCAAGACCAAAACTAAGAAGTCTAGTCCTCAAGACACGC***ATCCAAACAGT*CAATGCCATTAA**

>intergenic 1538…994 Red = MITE,

**GTTAAG**CAGAAATACGTTTTTTTTGTAGGGTGGGTAGAGCAGAGCGAAACCCACCAAACCTTTGAAATTTATAACAATGGTGGGTTGGGCGTCCCATAACGATGTTGCGCGGGCAACCACAAGGGATTGCCCCTACCCACCCTACAAAAAAAC**CTTAACTTAATGGCATTG*ATCCAAACAGT***GCCGTTGAACTGTTTACCACTTTTGGCCGCGACATTGGTCTCCATAAACTTGGCAAAAGCGAAGGCAATCTACTGAGTTACGGTGCTTTTTTCCATCCTGAAAAATGGCAACCTCCGTTTAACAAGCGCCATTGCTTACTTGCACCGGGTTTTTATAATGCCAAAAGCGGCACGATAGAAACTTTTTCTTCAGCAGACATCAAAGAACATCTCAAATATTCTTGGTATCGAGGTAAAGATAGTGGCTCCCATCCTTGGGAAGCTGATACCATTCCCAATTATGCCCCAGAGTCTGAAAAATACAGTTGGGCAAAAGCCCCTAGATACAAGGACACCGTAGTAGAAGTTGGACCATTGGCAGAA

>2601783458 Nickel-dependent hydrogenase [Thiomargarita nelsonii bud S10 Ga0063879 : Ga0063879_1132] (-)strand 499..993 (-)

ATGGTCGTTGCTGGCGATCCATTGATAACCGATTTTTTCAAACAAGAAGGCTCCAATAGTTGGCTGCGTCAATTCAGCCGCCTCCATCGTCCGGTTATAACCATGAAACTGATGCGGGAAACGTTCAAAGAAATTTTGAAAAATATCAATGAACCGTTTTACCAAGAACCCTCAACACTGGGAGATGGCGAAGGTGAAGGACTAATACAGGCAGCGAGGGGATGTTTAGGACATTGGGTTAAATTCAAAAACAATCAAATTGAGAAATATCAGATTATCACCCCAACGGCTTGGAATGCGTCCCCAAGGGATTCTGATGATAAACTTGGACACTGGGAAAAAACCCTAGTTGGTACTGAAATTCGTGATATTGAAAATCCTGTGGAAGTTGGGCATATTATTCGCTCCCACGATGCCTGCCTTGTGTGTACCGTTCACTTTTTGGAGACGGACAAAAAGGTACGGTTTCATTTGCCGGGGGCTAGTATGAGTTAG
