# Supplementary material for: Single-Cell (Meta-)Genomics of a Dimorphic Candidatus Thiomargarita nelsonii Reveals Genomic Plasticity
Source: Front Microbiol. 2016 May 3;7:603. doi: 10.3389/fmicb.2016.00603 (PMC4853749; doi:10.3389/fmicb.2016.00603)
Supplement: Supplementary Material 6 — Table of RepeatModeler generated consensus sequences (.docx). [file DataSheet4.DOCX]

**Supplementary Table 1: Summary of BLASTN results of RepeatModeler consensus sequences against the genome of *Candidatus* Thiomargarita nelsonii Hydrate Ridge Bud S10.**

| Family Number | Length  (bps) | RM Hits | Contigs  With  BLASTN  Hits | Comments |
| --- | --- | --- | --- | --- |
| 24 | 220 | 22 | 150/439 | Contains hairpin loops and homologues occur broadly across the genome with annotations both in hypothetical genes, as well as, intergenic space. No hits NCBI CDD. |
| 26 | 2294 | 21 | 141/439 | Top BLASTN hits with hypothetical genes with CHAT domains and tetratricopetide repeat elements. Homologues occur broadly across the genome, esp. to bps between bases 593…653 which often is intergenic. NCBI CDD hits indicated tandem TPR_10 (pfam_13374) and tandem CHAT superfamily domains (pfam12770) as well. CHAT domain containing genes include caspases. |
| 32 | 3179 | 55 | 100/439 | Group II introns and their remnants, many annotated by IMG. Motifs include reverse transcriptase, HNH endonuclease, and IMG detected Group II catalytic region. |
| 35 | 1350 | 15 | 141/439 | BLASTN hits with hypothetical genes with leucine-rich repeats, CRISPR elements, etc… |
| 176 | 493 | 37 | 303/439 | Contains palindromic regions, intergenic and commonly associated with Family 318. |
| 318 | 1015 | 17 | 210/439 | Contains palindromic regions, commonly associated with Family 176, occurs broadly across genome esp. bps between 1986…2026. Top BLASTN hits with P-type ATPases. NCBI Conserved Domain hit (bases 286…411, E-value 9.80e^-14^) was Eukaryotic RNA polymerase I subunit RPA34.5 and a Cation transport ATPase (COG2217, bases 572…739, 5.20e^-18^) |
| 325 | 2133 | 28 | 144/439 | Top BLASTN hits (e-value = 0) hits multiple hypothetical genes and the intergenic space around them but one gene was annotated as DDE domain transposase (pfam01609). The top NCBI CDD hit (bases 1878..s2054, E-value1.27e^-10^) was Eukaryotic RNA polymerase I subunit RPA34.5. |
| 867 | 1808 | 15 | 38/439 | Top BLASTN hits (e-value = 0) hits with an IS605 insertion element. |
